# *Paracoccidioides brasiliensis* presents metabolic reprogramming and secretes a serine proteinase during murine infection

**DOI:** 10.1080/21505594.2017.1355660

**Published:** 2017-07-13

**Authors:** Laurine Lacerda Pigosso, Lilian Cristiane Baeza, Mariana Vieira Tomazett, Mariana Batista Rodrigues Faleiro, Veridiana Maria Brianezi Dignani de Moura, Alexandre Melo Bailão, Clayton Luiz Borges, Juliana Alves Parente Rocha, Gabriel Rocha Fernandes, Gregory M. Gauthier, Celia Maria de Almeida Soares

**Affiliations:** a Laboratório de Biologia Molecular, Instituto de Ciências Biológicas, Universidade Federal de Goiás, Campus Samambaia s/n, Goiânia, Goiás, Brazil; b Laboratório de Patologia, Escola de Veterinária e Zootecnia, Universidade Federal de Goiás, Campus Samambaia s/n, Goiânia, Goiás, Brazil; c Instituto René Rachou, Fundação Oswaldo Cruz, Belo Horizonte, Minas Gerais, Brazil; d Department of Medicine, University of Wisconsin, Madison, WI, USA

**Keywords:** metabolic adaptation, *Paracoccidioides brasiliensis*, proteome, serine proteinase, transcriptome

## Abstract

*Paracoccidoides brasiliensis* and *Paracoccidioides lutzii*, the etiologic agents of paracoccidioidomycosis, cause disease in healthy and immunocompromised persons in Latin America. We developed a method for harvesting *P. brasiliensis* yeast cells from infected murine lung to facilitate *in vivo* transcriptional and proteomic profiling. *P. brasiliensis* harvested at 6 h post-infection were analyzed using RNAseq and LC-MS^E^. *In vivo* yeast cells had 594 differentially expressed transcripts and 350 differentially expressed proteins. Integration of transcriptional and proteomic data indicated that early in infection (6 h)*, P. brasiliensis* yeast cells underwent a shift in metabolism from glycolysis to β-oxidation, upregulated detoxifying enzymes to defend against oxidative stress, and repressed cell wall biosynthesis. Bioinformatics and functional analyses also demonstrated that a serine proteinase was upregulated and secreted *in vivo*. To our knowledge this is the first study depicting transcriptional and proteomic data of *P. brasiliensis* yeast cells upon 6 h post-infection of mouse lung.

## Introduction

*Paracoccidioides brasiliensis* and *Paracoccidioides lutzii*, the etiological agents of paracoccidioidomycosis (PCM), are fungal pathogens that are acquired following the inhalation of mycelia fragments or conidia into the lungs of a human host.^1^ A defining feature of *Paracoccidioides* pathogenesis is the morphologic transition from mycelia or conidia to yeast cells, which is governed by temperature and occurs when mycelia and conidia enter the lungs*. Paracoccidioides* causes a wide range of clinical manifestations, which range from isolated lung infection to deep-seated, life-threating infection, such as disseminated PCM, which occurs through hematogenous spread.^2,3^

Our goal is to characterize genes involved with the adaptive response of *P. brasiliensis* during the early stages of pulmonary infection. Although identification of these genes is crucial for the understanding of fungal pathogenesis, the limited abundance of *P. brasiliensis* yeast cells at the sites of infection, makes gene and protein assays challenging. To overcome the limited availability of fungal material, *in vitro* and *ex vivo* previous approaches have been used to mimic conditions of infection. Prior studies have profiled gene expression in *P. lutzii* yeast cells incubated in human blood at 36°C, using cDNA representational difference analysis (cDNA-RDA), as well as *P. brasiliensis* yeast cells internalized by macrophages, using microarray technology.^4,5^ These studies demonstrated down regulation of transcripts related to cell wall metabolism and glycolysis, as well as upregulation of putative virulence factors, such as a serine protease. Approaches used to identify genes relevant for infection at the proteomic level, include the use of various *in vitro* experimental conditions such as iron deprivation, hypoxia, oxidative, nitrosative stress, and fungal-macrophage interactions.^6,7,8,9,10^ The later approach demonstrated that during macrophage infection, yeast cells remodel their metabolism to recycle carbon containing molecules and induce gluconeogenesis. This metabolic remodeling may play an important role in the adaptive response to phagocytosis. Additionally, during macrophage infection, *P. brasiliensis* yeast cells induce the expression of cytochrome C peroxidase (CCP), an anti-oxidant molecule and potential virulence factor. Antisense CCP knockdown strains have reduced survival upon macrophage interaction and during infection in BALB/c mice. Additionally, mutants with low CCP expression were more sensitive to oxidative and nitrosative stress.^9,10^

While *in vitro* and *ex vivo* studies have provided insight on fungal processes involved with murine pulmonary infection, *in vivo* analysis of *P. brasiliensis* yeast cell gene expression during pulmonary infection has not been conducted. To understand how *P. brasiliensis* adapts to the host, we established a model of intranasal infection coupled with bronchoalveolar lavage for large-scale *in vivo* gene and protein expression analyses during pulmonary infection. Thus, we captured, for the first time, large-scale *in vivo* gene expression during invasive PCM. Because transcriptome analysis provides a limited understanding of the biology associated with *in vivo* survival, we also investigated the underlying proteomic changes during infection. For transcriptome and proteome analyses, we focused our attention at 6 hours post-inhalation because our data demonstrated that at this time point, *P. brasiliensis* invaded mouse lung tissue. Our profiling studies paint a picture of lung invasion in which *P. brasiliensis* alters the expression of genes related to several functional categories including energy metabolism and cell wall metabolism. Some active processes presumably help the fungus to survive in lung, as here identified, such as the accumulation of detoxifying enzymes. On the basis of our results, we propose that *P. brasiliensis* remodels cellular lipid metabolism to catabolize its own lipid stores via β oxidation, glyoxylate cycle, which were strongly induced during the first 6 hours of lung infection. Moreover, our approach identified a secreted serine proteinase that may facilitate invasion of lung tissue and dissemination of *P. brasiliensis* to other organs.

## Results

### Establishing a method for infection and recovery of yeast cells *in vivo*

**Figure 1.**
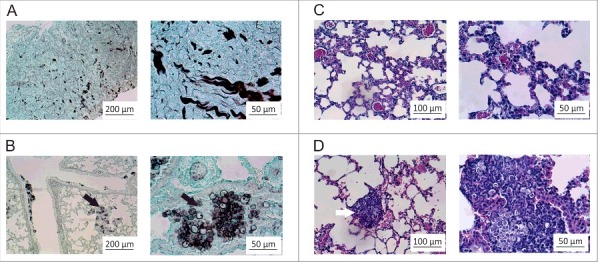
Histopathological images of lung sections. The animals were killed 6 h post-intranasal infection and the lungs were harvested for histopathologic analysis. (A) Gomori-Grocott staining of lung slices of control animals treated with saline solution (NaCl 0.9%) (B) Gomori-Grocott staining of lung slices of animals at 6 h post infection. Black arrows point toward *P. brasiliensis* yeast. (C) Hematoxylin-Eosin (HE) staining in lung slices of control animals (D) HE staining of lung slices of animals at 6 h post-infection. Inflammatory polymorphonuclear cells surround *P. brasiliensis* yeast (white arrows).

Transcriptomic and proteomic approaches can identify genes and biologic processes important for infection. Although *in vivo* profiling using mouse models have the potential to uncover gene and protein expression during infection, it has remained a challenge to obtain sufficient quantity of yeast cells for RNA and protein analysis. To overcome this barrier, we sought to establish an infection model and method for recovering sufficient quantity of yeast cells for RNA and protein extraction. For this, mice were infected with *P. brasiliensis* by intranasal inhalation of yeast cells and after 6 h we performed extensive lung lavage to harvest yeast cells in the bronchoalveolar fluid. We validated our infection model by analyzing lung sections of infected mice at different post-infection time points. At 6 and 24 h after intranasal exposure, mice were killed and lung tissue collected for histological processing. The experiments were conducted in biologic (3 animals) and experimental (3 time-independent experiments) triplicates. As observed in Fig. 1A and C, lungs of control group, inoculated with saline solution (NaCl 0.9%), had a normal histomorphology including terminal bronchioles, surrounding muscle tissue, and the alveolar duct structure. The lung tissue sections were stained with Gomori-Grocott^11^ to highlight the presence of fungal cells (Fig. 1B). Staining with hematoxylin-eosin^12^ was used to evaluate for inflammation (Fig. 1D). Histopathological examination of lung sections at 6 h post-infection showed neutrophilic inflammatory infiltrate associated with circular birefringent fungi cells, ranging from 6 to 20 µm in diameter (Fig. 1D; Fig. S1).

Figure S2 depicts mouse lungs at 24 hours post infection. Histopathology at 24 h post-infection showed a large increase in neutrophilic inflammatory infiltrate associated with circular birefringent fungal cells (Fig. S2C and 2D). Since we were interested in the initial stages of infection, the 6 h time point was selected for harvest of *P. brasiliensis* because there were abundant yeast cells in the lungs. Transcriptional and proteomic analysis was performed using high-throughput RNA Illumina sequencing (RNAseq) and NanoUPLC-MS^E^, respectively.

The procedure for obtaining bronchoalveolar lavage was validated using histopathological analyzes and evaluation of yeast cells number, as depicted in Fig S3. After counting of 50 fields, an average of 106 and 30 yeast cells was observed in lungs that were not submitted to bronchoalveolar lavage and lungs after bronchoalveolar lavage, respectively (Fig S3). Counting of yeast cells in the bronchoalveolar material provided very similar results (data not shown).

### Investigating the transcriptome of *Paracoccidioides brasiliensis* yeast cells at 6 h post-infection

On the basis of our pilot experiments, we selected the 6 h post-infection time point for transcriptome analysis. cDNA libraries were generated and the sequenced reads were mapped to the reference *Paracoccidioides* genome (Pb18), (http://www.broadinstitute.org/annotation/genome/paracoccidioides_brasiliensis/MultiHome.html) using the Bowtie 2 tool (doi:10.1038/nmeth.1923) and analyzed by DEGseq package.^13^ The list of differentially expressed genes (DEGs) was obtained by comparing RNA levels of yeast cells at 6 h post-infection to the *in vitro* control; (control fungal cells were obtained by incubation of *P. brasiliensis* cells in BHI liquid medium at 37°C during 48 h). To determine the up- and downregulated transcripts, a cut-off of 2.0-fold change was applied. Table S1 lists the differentially induced transcripts and Table S2 lists the differentially repressed transcripts.

Five hundred and ninety four differentially expressed transcripts were identified from yeast cells at 6 h post-infection. From this total, 73.40% of transcripts were functionally annotated, corresponding to 436 genes, and 26.60% were unclassified transcripts corresponding to 158 genes.

We used the functional catalog (FunCat) for functional enrichment analysis. We additionally included KEGG terms, when relevant. Subcategories of metabolism related to amino acid, nitrogen/sulfur, C-compound/carbohydrate, lipid/ fatty acid, purines, secondary, and phosphate metabolisms were regulated in host-pathogen interaction (Tables S1 and S2). Categories associated with cell cycle/DNA processing, transcription, protein synthesis and fate and cellular transport were also largely represented in the transcriptome.

The functional classifications and the percentage of up- and downregulated transcripts in each classified category are shown in Fig S4. Functional enrichment analysis demonstrated that at 6 h post-infection of mouse lung, most of the DEGs in *P. brasiliensis* corresponded to downregulated categories (Table S1 and 2, Fig S4). A total of 430 DEGs (72.6%) were repressed and 154 DEGs (27.4%) were induced at 6 h post-infection. The majority of enriched categories for upregulated DEGs were related to metabolism and cellular transport. The majority of enriched categories for downregulated DEGs were metabolism, transcription and protein synthesis.

### Changed expression connected to upregulated transcripts

Figure S4 depicts enriched categories for upregulated DEGs. Metabolism and cellular transport constitute the functional categories most highly enriched during fungal infection. In the metabolism category, the oxidation of fatty acids was the most enriched pathway, which includes acyl-CoA dehydrogenases (PADG_07604, PADG_05046, PADG_06335), short chain dehydrogenases/reductases (PADG_06247, PADG_06006), carnitine transport/metabolism (PADG_05773, PADG_06378), peroxisomal hydratase-dehydrogenase-epimerase (PADG_08651) and a 3-hydroxybutyryl-CoA dehydratase (PADG_05039). These genes demonstrated increase transcript abundance that ranged from 2.29 to 17.00 (Table S1). In addition, ethanol producing genes were induced including alcohol dehydrogenase (PADG_04701) and aldehyde dehydrogenase (PADG_05081) with 4.38 and 5.13-fold change, respectively. As depicted in Table S1, genes involved with cellular transport including amino acid permease (PADG_11786), MFS drug transporters ( PADG_08041, PADG_07610, PADG_04733), multidrug transporter (PADG_12014), ), ABC drug transporter (PADG_06245) and zinc-regulated transporter (PADG_06417), were among the transcripts induced *in vivo*.

Table 1 depicts the most induced transcripts (≥ 4-fold) in yeast cells infecting mouse lungs. The most induced transcripts, depicted in Table 1, include those related to degradation of fatty acids and carbohydrates, nitrogen and ethanol metabolism, proteins related to the defense to stress, among others.

**Table 1.** The most upregulated transcripts *in vivo* at 6-hours post-infection.

Accession number^a^	Gene Description^b^	Ratio Infection/Control^c^	Function^d^
PADG_06490	Formamidase	22.77	Nitrogen metabolism
PADG_05773	Carnitinyl-CoA dehydratase	17.00	Lipid, fatty acid and isoprenoid metabolism
PADG_07604	Acyl-CoA dehydrogenase	13.46	Fatty acid degradation
PADG_08544	GABA permease	10.80	Transport
PADG_07751	Mitochondrial integral membrane protein	8.35	Transport
PADG_05876	Centromere/microtubule-binding protein Cbf5	8.22	Cell cycle
PADG_06196	12-oxophytodienoate reductase	7.43	Energy conversion and regeneration
PADG_07803	60S ribosomal protein L12	6.58	Protein synthesis
PADG_08651	Peroxisomal hydratase-dehydrogenase-epimerase	6.02	Multifunctional β-oxidation protein
PADG_12014	Pleiotropic ABC efflux transporter of multiple drugs	5.84	Transporters
PADG_08034	Dienelactone hydrolase family protein	5.58	Secondary metabolism
PADG_07809	Cysteine protease PalB	5.56	Protein/peptide degradation
PADG_06309	Glucose-methanol-choline oxidoreductase	5.37	Energy conversion and regeneration
PADG_05757	Sugar transporter	5.26	Transport
PADG_06197	Xanthine dehydrogenase, molybdopterin binding subunit	5.24	Purine metabolism
PADG_05081	Aldehyde dehydrogenase	5.13	Fermentation
PADG_04907	Chaperone/heat shock protein	5.07	Stress response
PADG_06425	Delta(3,5)-Delta(2,4)-dienoyl-CoA isomerase	4.99	Transport
PADG_06477	Phosphotyrosine protein phosphatase	4.70	Signal transduction
PADG_06322	Cytochrome c peroxidase, peroxissomal	4.68	Stress response
PADG_04543	Non-specific lipid-transfer protein	4.51	Transport
PADG_07450	Ubiquitin-conjugating enzyme	4.48	Protein/peptide degradation
PADG_04701	Alcohol dehydrogenase	4.38	Fermentation
PADG_07732	Aspartate-tRNA(Asn) ligase	4.22	Protein synthesis
PADG_08012	Fructose-bisphosphate aldolase	4.16	Glycolysis and Gluconeogenesis
PADG_07783	Phosphotransferase enzyme family domain-containing protein	4.04	Protein modification

Notes.

a Identification of differentially regulated genes from *Paracoccidioides* genome database (http://www.broadinstitute.org/annotation/genome/paracoccidioides_brasiliensis/MultiHome.html) using the statistical package DEGSeq, the Bioconductor, using Fisher's test implemented in the package.

b Genes annotation from *Paracoccidioides* genome database or by homology from NCBI database (http://www.ncbi.nlm.nih.gov/)

c Infection/Control means: The level of expression in yeast cells derived from infected lung divided by the level in the control yeast cells.

d Biological process of differentially expressed proteins from MIPS (http://mips.helmholtz-muenchen.de/funcatDB/) and Uniprot databases (http://www.uniprot.org/).

### Changed expression connected to downregulated transcripts

As described above, the majority of DEGs were down-expressed *in vivo* compared with the *in vitro* control. A number of downregulated genes were involved in electron transport and membrane-associated energy conservation (Table S2). This category contains 10 downregulated genes, comprising several subunits of NADH-ubiquinone oxidoreductase (PADG_04699, PADG_08216), ATP synthase complex, and cytochrome complex (Table S2).

Three genes involved in the TCA cycle including malate dehydrogenase (PADG_07210), ATP citrate (pro-S)-lyase (PADG_04994), pyruvate dehydrogenase complex dihydrolipoamide acetyltransferase (PADG_07213), were downregulated (Table S2).

**Figure 2.**
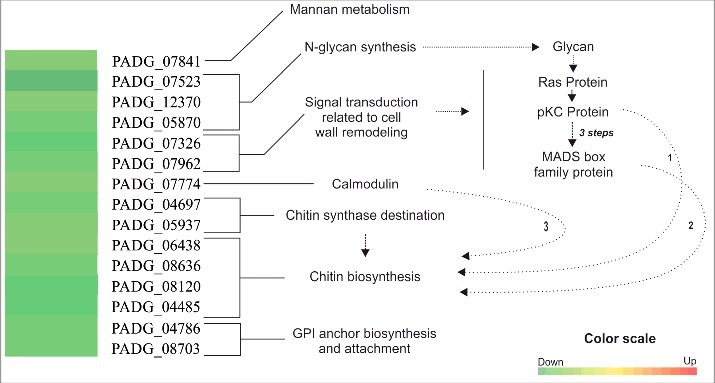
Schematic representation of downregulated transcripts related to *P. brasiliensis* cell wall homeostasis. Transcripts related to mannan metabolism was induced (PADG_04693) or repressed (PADG_07841). Transcripts encoding proteins related to glycan synthesis (PADG_07523, PADG_12370 and PADG_05870) were downregulated upon 6 h post- infection in mouse lung. Down-regulation of glycan synthesis can be triggered by downregulation of the Ras-dependent signaling pathway (PADG_07962) which activates chitin biosynthesis (indicated by arrow 1). Also, protein kinase C (pKC, PADG_07326) and calmodulin (PADG_07774) dependent signaling pathways can modulate chitin synthesis (indicated by arrows 1 and 3, respectively). Both are negativelly regulated in this condition. Transcripts encoding proteins related to chitin biosynthesis (PADG_06438, PADG_08636, PADG_07802, PADG_08120 and PADG_04485) were repressed; (PADG_12130) was induced. The downregulation of the cell wall synthesis is also evidenced by the negative regulation of transcripts encoding proteins related to the cellular destination of chitin synthase (PADG_04697, PADG_05937)and GPI anchoring (PADG_08703). Transcripts encoding proteins related to GPI synthesis wasrepressed (PADG_04786). Thoese transcripts are supplied in Tables S1 and 2. The MultiExperiment Viewer software V.4.8 (www.tm4.org/mev/) was used to group the compare data of expression ratios and color scale was adapted.

The metabolic processes involving the cell wall were downregulated in yeast cells at 6 h post-infection (Table S2). A total of 25 trancripts related to cell wall metabolism were identified that were downregulated. Three transcripts encoding proteins related to glycan metabolism such as α 1,3 glucosidase (PADG_07523); endo-1,3(4)-β-glucanase (PADG_12370) and glucan synthesis regulatory protein (PADG_05870) were downregulated with expression ratios from 0.37 to 0.1 relative to the *in vitro* control (Table S2). The activation of the β-1,3 glucan synthase is regulated by Ras/Rho GTPases cell signaling pathway, that includes protein kinase C and MADS family protein.^14^ As illustrated in Fig. 2, this pathway was downregulated at 6 h of *P. brasiliensis* post-infection infection in mouse lung (PADG_07326: serine/threonine-protein kinase PRKX and PADG_07962: MADS box transcription factor Mcm1), which could be related with lower glycan content in yeast cells upon infection. Also, the proteins pKC and calmodulin are described as activators of chitin synthesis^15^ and, interestingly, both signaling pathways can be repressed, putatively leading to repression of chitin synthesis. Transcripts related to chitin synthesis (PADG_06438, PADG_08636, PADG_08120 and PADG_04485) were downregulated *in vivo* with expression ratios 0.1 – 0.45 relative to the *in vitro* control (Table S2). In addition, chitin activators (PADG_04697 and PADG_05937), responsible for localization of chitin synthases to the cell wall, were negatively regulated, which has been described in other fungal models.^16^ In addition, transcripts related to GPI synthesis (PADG_04786: GPI ethanolamine phosphate transferase) and attachment of GPI anchors into proteins (PADG_08703: GPI transamidase component GPI16) were also downregulated *in vivo* (Table S2).

The metabolism category includes primary metabolism of amino acids, from which the metabolism of tyrosine and phenylalanine was especially downregulated. The category of mitotic cell cycle and cell cycle control was enriched with 21 downregulated genes including predicted phosphatases and cell division control proteins (Table S2). Regarding DNA processing, genes in this category were downregulated *in vivo*. In this category there were 11 proteins important for DNA duplication such as ATP-dependent DNA helicase II , replication factor-A protein (PADG_07835), DNA replication licensing factors (PADG_08558, PADG_07901), DNA topoisomerases PADG_07812, PADG_04954, PADG_06339), DNA-directed RNA polymerases (PADG_06985, PADG_08395, PADG_07655), FACT complex subunit pob3 (PADG_07672), nucleosome assembly protein 1-like 1 (PADG_05893) , and 5 transcripts encoding proteins/enzymes of DNA repair (Table S2).

The categories of protein synthesis and degradation comprised a large number of genes with reduced expression (Table S2). In the category of protein synthesis, 60 downregulated genes were present including those related to ribosomal biogenesis and translational machinery.

Transcriptional control depicts several downregulated genes (Table S2). Surprisingly, the gene encoding the HapX transcription factor (PADG_07492), involved in the homeostasis of iron is among the most repressed in yeast cells at 6 h post-infection in mouse lung. Other repressed transcription factor *in vivo* includes APSES MbpA (PADG_07224) involved in the regulation of morphogenesis including filamentation and differentiation in *Candida albicans* and *Aspergillus fumigatus*.^17^

Signal transduction category had 25 downregulated genes (Table S2) involving several pathways such as calcium-calmodulin, histidine kinase, MAP kinase. Different protein kinases are among the most repressed genes in *P. brasiliensis* yeast cells at 6 h post-infection in mouse lung (Table S2).

### Quantitative proteome profiles of yeast cells infecting mouse lungs

#### Proteomic data quality analysis

Protein quantification was performed using NanoUPLCMS^E^ as described previously.^18,19,20^ The resulting NanoUPLC-MS^E^ protein and peptide data generated by the PLGS process are shown in Figs. S5, S6 and S7. The false positive rates of proteins obtained from yeast cells at 6 h post-infection of mouse lung and from *in vitro* control were 0.99% and 0.67%, respectively. These experiments resulted in 9,787 and 17,537 identified peptides, where 52 and 58% were obtained from peptide match type data in the first pass and 9% (for both control and test) from the second pass^20^ for *in vivo* and *in vitro* yeast cells, respectively (Fig S5). A total of 13% and 21% of total peptides were identified by a missed trypsin cleavage in control and infection conditions, respectively, whereas an in-source fragmentation rate of the same 7% from control and 11% in yeast cells at 6 h post-infection of mouse lung was obtained (Fig. S5).

Figure S6 shows the peptide parts per million error (ppm) indicating that the majority, 63.19% and 72.56%, of identified peptides were detected with an error of less than 5 ppm for yeast cells isolated from mouse lung and *in vitro* control, respectively. For error of less than 10 ppm, 87.75% and 92.22% peptides were identified for yeast cells infecting mouse lung and control, respectively. Fig S7 depicts the results obtained from dynamic range detection indicating that a 3-log range concentration and a good detection distribution of high and low molecular weights were obtained for the both conditions.

### The pattern of proteins differentially accumulated upon *P. brasiliensis* infection of mouse lung

The 3 parallel independent infection assays were performed and equimolar amounts of protein extracts were mixed and used in proteomic analysis. Pooled protein extracts were run in 3 technical replicates and only proteins found in 2 out of the 3 technical replicates, by an identical set of at least 2 unique tryptic peptides, were considered for further analysis. Those criteria have been extensively used in label-free MSE proteomic assays.^21,22,23^

Five hundred and 8 proteins were identified, after filtering, using the criterion of minimum repeat rate of 2. A 1.5-fold change was used as a threshold to determine differentially expressed proteins. 147 and 203 proteins down and upregulated, respectively, *in vivo*, versus *in vitro* control (Tables S3 and S4) were obtained after filtering using the parameters above. Biological processes and the percentage of down and up- regulated proteins in each classified category are depicted in Fig. S8. The identified proteins were classified on the basis of their function according to the Munich Information Center for Protein Sequences (MIPS) categorization.

### Proteins downregulated in yeast cells during mouse infection

Proteomic analysis of *in vivo* yeast cells demonstrated that proteins involved with amino acid biosynthesis and protein synthesis were decreased. Enzymes in biosynthetic pathways for threonine, tryptophan, glutamate, and glycine were reduced including threonine synthase (PADG_02777), anthranilate phosphoribosyltransferase (PADG_01789), glutamate decarboxylase (PADG_06319) and serine hydroxymethyl transferase (PADG_05277), respectively. In addition, 30 proteins involved with protein synthesis were decreased (Table S3).

### Proteins upregulated in yeast cells during mouse infection

*P. brasiliensis* proteins upregulated *in vivo* were involved with multiple pathways including fatty acid catabolism (beta and omega oxidation), glyoxylate cycle, pentose-phosphate pathway, and ethanol production (Table S4). Proteins involved with fatty acid breakdown, acyl-CoA-binding protein (PADG_01363), enoyl CoA hydratase (PADG_01209), and isocitrate lyase (PADG_01483) in the glyoxylate cycle was induced 1.53-fold *in vivo*. Enzymes involved with ethanol production are upregulated; alcohol dehydrogenase (PADG_04701) and pyruvate decarboxylase (PADG_00714) increased 13.73 and 1.58 fold compared with *in vitro* control, respectively.

In the functional class of protein synthesis, 39 molcules were increased (Table S4). They belong to the subcategories of ribosome biogenesis, translation, translational control and translational initiation. Thirty-two were induced in the functional class of protein degradation (Table S4), including components of the proteasome complex, ubiquitin system and proteases. The up regulation of enzymes involved with protein degradation, may supply anaplerotic precursors such as pyruvate, which can be metabolized by alcohol dehydrogenase (PADG_04701) and pyruvate decarboxylase (PADG_00714), as cited above.

**Figure 3.**
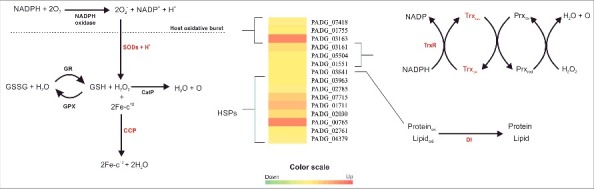
Overview of detoxification mechanisms against oxidative stress in *P. brasiliensis*, during lung infection. This figure summarizes data from proteomic analyses and suggests the mechanism used by this fungus in response to oxidative stress. SOD: superoxide dismutase (PADG_07418, PADG_01755); Cat P: peroxissomal catalase; GR: Gluthatione reductase; GPX: Gluthatione peroxidase; CCP: Cytochrome c peroxidase (PADG_03163); TrxR: Thioredoxin reductase (PADG_01551); Trx: Thioredoxin (PADG_03161 and PADG_05504); DI: disulfide isomerase (PADG_03841); HSP: Heat shock protein (PADG_03963, PADG_02785, PADG_07715, PADG_01711, PADG_02030, PADG_00765, PADG_02761 and PADG_04379). SODs mediate the detoxification of superoxide to hydrogen peroxide. Degradation of hydrogen peroxide is catalyzed by catalase and alternative pathways, for example, via gluthatione and cytochrome c peroxidase. The MultiExperiment Viewer software V.4.8 (www.tm4.org/mev/) was used to group the compare data of expression ratios and color scale was adapted.

Fifteen proteins predicted to be involved with cell rescue, defense and virulence were increased in yeast cells infecting mouse lung. Several heat shock proteins including 30 kDa (PADG_03963), hsp88 (PADG_02785), HSP90 (PADG_07715), Hsp90 co-chaperone AHA1 (PADG_01711), hsp90 co-chaperone Cdc37 (PADG_02030), hsp98 (PADG_00765), SSB1 (PADG_02761), and STI1 (PADG_04379) were increased. Fig. 3 presents an overview of the response to oxidative stress, as suggested by the proteomic analysis. Several proteins that have been described as virulence factors in other pathogenic microorganisms were upregulated in *P. brasiliensis* yeast cells after 6 h of lung infection. These include proteins involved with detoxification of phagocyte-derived oxidative stress such as two superoxide dismutases, Cu/Zn-containing SOD1 (PADG_07418) and Fe/Mn-containing SOD2 (PADG_01755), cytochrome C peroxidase (PADG_03163), three thioredoxins (PADG_03161, PADG_05504, PADG_01551), and disulfide isomerase (PADG_03841).

### Overview of metabolic changes in *P. brasiliensis* at 6 h post-infection in mouse lung

**Figure 4.**
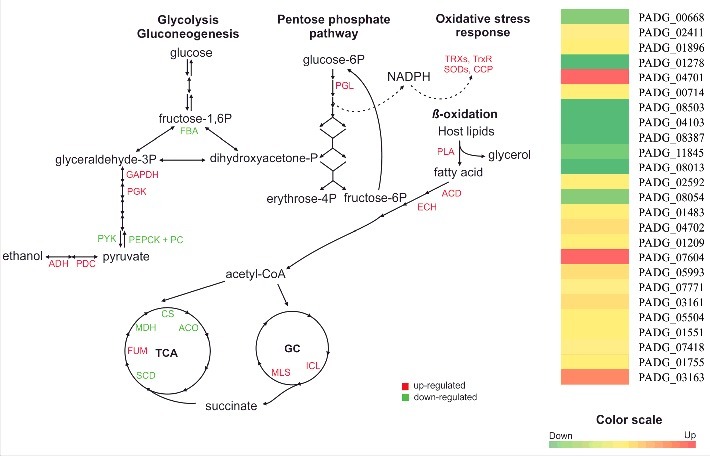
Adaptive metabolic response of *P. brasiliensis* during lung infection. FBA fructose biphosphate aldolase (PADG_00668), GAPDH Glyceraldehyde 3-phosphate dehydrogenase (PADG_02411), PGK phosphoglycerate kinase (PADG_01896), PYK pyruvate kinase (PADG_01278), ADH alcohol dehydrogenase (PADG_04701), PDC pyruvate decarboxylase (PADG_00714), PEPCK PEP carboxykinase (PADG_08503), PC pyruvate carboxylase (PADG_04103), CS citrate synthase (PADG_08387), ACO aconitase (PADG_11845), SCD succinate dehydrogenase (PADG_08013), FUM fumarase (PADG_02592), MDH malate dehydrogenase (PADG_08054), ICL isocitrate lyase (PADG_01483), MLS malate synthase (PADG_04702), ECH enoyl-CoA hydratase (PADG_01209), ACD acyl-CoA dehydrogenase (PADG_07604) PLA phospholipase (PADG_05993), PGL phosphoglucolactonase (PADG_07771), TRX thioredoxins (PADG_03161 and PADG_05504), TrxR thioredoxin reductase (PADG_01551), SOD superoxide dismutase (PADG_07418 and PADG_01755), CCP cytochrome C peroxidase (PADG_03163). The MultiExperiment Viewer software V.4.8 (www.tm4.org/mev/) was used to group the compare data of expression ratios and color scale was adapted. To analyze the conditions, the up- and downregulated genes/proteins were integrated and the expression levels were used to build a heat map.

We have integrated transcriptional and proteomic data to summarize the fungal response after 6 h in mouse lung. Our data suggest that yeast cells reprogram pathways for energy metabolism (Fig. 4) including using lipids as an energy source during infection. Most of the enzymes involved in oxidation of even and odd chain fatty acids were upregulated. Similarly, enzyme in pentose phosphate pathway was increased, which can supply the redox equivalents for the oxidative stress response. Glyoxylase was also induced, which detoxifies methylglyoxal generated from lipid peroxidation. The glyoxylate cycle was upregulated, which further supports lipid catabolism during infection. Interestingly, transcripts and proteins related to aerobic metabolism, which supports fatty acid derived energy production were downregulated. This metabolic contradiction may be explained by the fact that yeast cells were grown in a rich medium with abundant nutrients and then transferred to a hostile *in vivo* environment. Thus, the high metabolic activity that is required to support the exponential growth phase *in vitro* is no longer required during early phases of infection, when the yeast cells are adapting to the *in vivo* environment. Rather, during infection *P. brasiliensis* utilizes acetyl- and propionyl-CoA, which are derived from fatty acid catabolism, to fuel glyoxylate cycle for anaplerosis of oxaloacetate, which provide building blocks for biomolecule synthesis.

### Immunohistochemical analysis of infected mice lungs with polyclonal antibodies to serine protease (PADG_07422)

**Figure 5.**
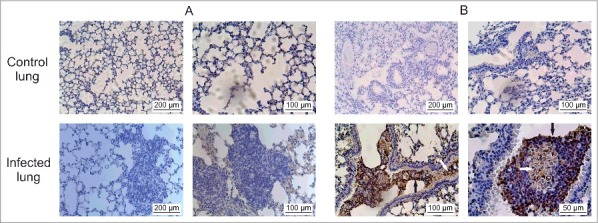
Histopathological images of lung sections at 6 h post-infection. (A) In situ presence of enolase, a control protein. (B) The presence of serine proteinase during infection in lung tissue. White arrows evidence fungal cells and black arrows evidence the labeling occurring also outside fungal cells. Scale bars are indicated. The polyclonal antibodies anti-enolase^25^ at 1:150 dilution and anti-serine-proteinase^24^ at 1:150 dilution, previously obtained, were used in A and B, respectively. The chromogen 3.3′ diaminobenzidine tetrahydrocloride (DAB, Dako, #K3468-1) was used, and sections were then counterstained with Mayer's hematoxylin and examined by light microscopy.

During pulmonary infection, *P. brasiliensis* yeast cells upregulated 32 proteins involved with protein fate and degradation (Table S4). In this class of proteins we prioritized a serine proteinase (PADG_07422), which was 2.4-fold upregulated, for *in vivo* analysis by immunostaining. Previous studies in our laboratory demonstrated that PADG_07422 is a secreted serine proteinase and is induced during nitrogen deprivation.^24^ After 6 h of intranasal infection, polymorphonuclear inflammatory infiltrate surrounding *P. brasiliensis* yeast cells stained positive for the serine proteinase (Fig. 5B). In addition, the serine protease was also detected on the surface and in the cytoplasm of murine cells (Fig. 5B). The detection of enolase,^25^ which was used as a control, only stained yeast cells (Fig. 5A). Thus, the labeling of the host tissue supports that the serine protease is secreted by *P. brasiliensis* yeast *in vivo*.

**Figure 6.**
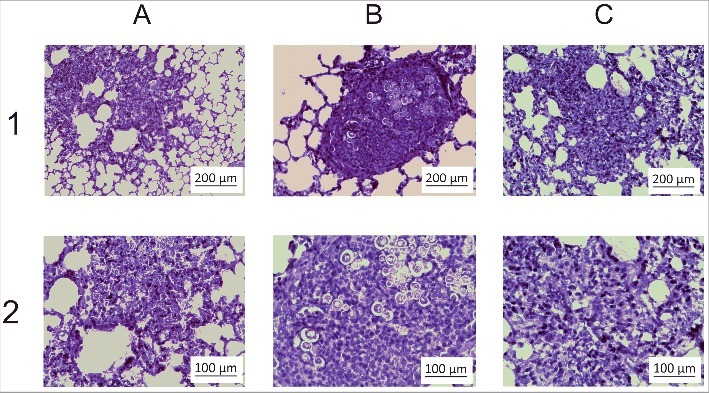
Histopathological analyzes of mice lung injected with immuno stimulants. After Lung tissues in (A) Carrageenan (B) Heat-killed yeast cells and (C) Zymozan injected mice. The presence of serine proteinase in the tissue was not detected. Scale bars are indicated. The polyclonal antibodies anti-serine-proteinase at 1:150 dilution was used. The chromogen 3.3′ diaminobenzidine tetrahydrocloride (DAB, Dako, #K3468-1) was used, and sections were then counterstained with Mayer's hematoxylin, and examined by light microscopy.

Additional controls were performed using different inducers of immune response to demonstrate lack of cross-reactivity of the anti-serine proteinase polyclonal antibodies to mouse proteins. Considering the inflammatory infiltrate caused by the inoculation of zymosan^26^, carrageenan^27^ or heat killed fungi, serine proteinase was not detected by immunohistochemistry assays, as shown in Fig. 6, panel A to C, respectively.

### Treatment of mouse with anti-serine proteinase polyclonal antibodies induces decreased fungal burden

**Figure 7.**
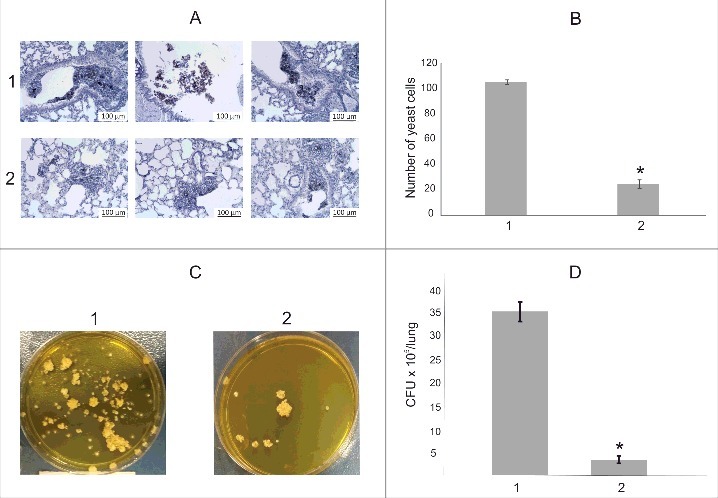
Treatment of mouse with polyclonal antibodies to serine proteinase (PADG_07422). (A) (B) Average counts of *P. brasiliensis* cells in lung tissue in 50 sections; control (1); animals pretreated with polyclonal anti-serine proteinase antibodies (2). (C) CFUs recovered after bronchoalveolar lavage of control (1) and animals treated with polyclonal antibodies (2). (D) CFUs counts for control (1) and pre-treated mouse (2). Asterisks evidence statistical differences observed by the Student T test presenting *p* value ≤ 0.05 were considered significant.

We performed an additional experiment to evaluate the potential role of serine proteinase (PADG_07422) in mouse infection by *P. brasiliensis*. After 6 hours of infection, the lungs of animals pretreated with anti-serine proteinase polyclonal antibodies and the control group were assessed using histopathological and quantitative analyses. The amount of fungus in the pre-treated animals was significantly reduced (Fig. 7 A), corresponding to approximately 23% of control, as depicted in Fig. 7 B. We also analyzed the CFU after bronchooalveolar lavage in infected, pre-treated mice (vs. control) with the polyclonal antibodies (Fig. 7 C). A 90.5% reduction in CFU was observed in the pre-treated animals.

### Increased production of ethanol and thiols by *P. brasiliensis* yeast *in vivo*

**Figure 8.**
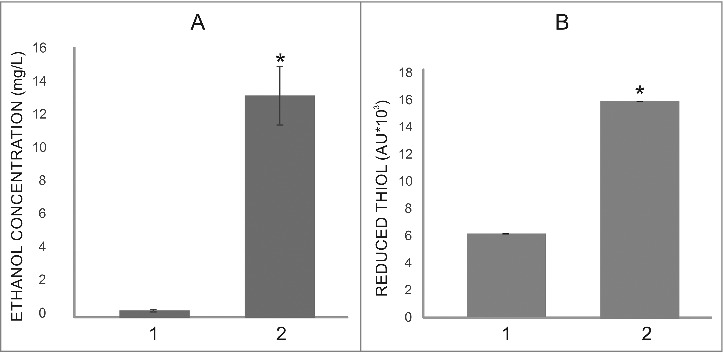
Ethanol and thiol measurements in protein extracts of yeast cells after 6 hours of mice infection. (A) Increase in ethanol production in yeast cells recovered from lung mice (1); control (2). (B) Thiol reduction level evaluated in yeast cells infecting mouse lung (1); control (2). Asterisks evidence statistical differences observed by the Student T test presenting *p* value ≤ 0.05 were considered significant.

At 6 h post-infection, 2 genes involved with ethanol production, pyruvate decarboxylase (PADG_00714) and alcohol dehydrogenase (PADG_04701) (Tables S1 and S4) were upregulated. Ethanol concentration was approximately 13-fold higher in yeast cells at 6 h of infection in mouse lung compared with controls (Fig. 8A).

Enzymes in the thioredoxin system are used to defend against oxidative stress. *In vivo* proteomics analyses demonstrated that two thioredoxins (PADG_03161 and PADG_05504) and a thioredoxin reductase (PADG_01551) were induced (Fig. 3, Table 4). To functionally assess fungal thioredoxin activity, we quantified thiol levels in yeast cells of *P. brasiliensis* at 6 h post-infection of mouse lung. Thiol levels were approximately 2.5-fold higher in yeast cells during infection (vs. *in vitro* control) (Fig. 8B).

## Discussion

In this study, we used transcriptional, proteomic and bioinformatics analyses to identify and characterize gene expression in *P. brasiliensis* during pulmonary infection. The proteomic and transcriptional experiments were designed to capture the fungal response early in the infection process. Studies focusing on host-microbe interactions at molecular level have traditionally been challenging due to the limited number of reliable methods for harvesting fungal cells from infected organs. Herein, we describe a straightforward method for recovering *P. brasiliensis* yeast cells during the early stages of pulmonary infection for *in vivo* transcriptional and proteomic analyses. Additionally, we integrated transcriptional and proteomic data to assess how *P. brasiliensis* adapts to the host during the initial stages of infection in mouse lung. The changes in proteins profiles showed low congruency with the transcripts levels. We attribute this fact to post transcriptional processes playing a critical role in the protein levels, as described in *Aspergillus flavus*.^28^ Although we have used a 2-fold threshold in the transcriptional analysis, the presence of excess of host RNAs in the samples, could impact the results.

Adaptive responses within 6 h of infection include inhibition of cell wall metabolism, and increase in pathways linked to protective responses against reactive oxygen species, as well as remodeling metabolism toward lipid degradation, ethanol production and glyoxylate cycle. In addition, a serine proteinase, potentially linked to pathogenesis was characterized.

Analysis of transcriptional data identified 1761 transcripts with ≈34% (594 genes) that were differentially expressed (164 upregulated, 430 downregulated) at 6 h post-infection. Analysis of the downregulated transcripts *in vivo* demonstrated a strong effect on cell wall metabolism. A striking pattern was the repression of several genes involved with the biosynthesis of glycan, chitin and genes that activate chitin synthases. Also, glycosyl hydrolases and chitin metabolism responsive genes were repressed. The cell wall is dynamic and responds to changes in the external environment. Multiple cellular functions, including cell wall remodeling, negatively regulated by the Zcf21 transcription factor, contribute to the ability of *C. albicans* to promote systemic infection.^29,30^ *Zcf21* mutants have higher content of cell wall polysaccharides, increased susceptibility to phagocytosis, and impaired intracellular survival in macrophages.^30^ If inhibition of cell wall genes in *P. brasiliensis* influences the host recognition remains to be elucidated.

We identified 350 proteins differentially expressed *in vivo* (203 upregulated, 147 downregulated). Among the upregulated proteins, an arsenal of ROS defense enzymes were found. Host phagocytic cells use reactive oxygen species as part of their armory to neutralize foreign microorganisms.^31^ The phagocytic cells of the innate immune system produce several oxidants that are presumed to play roles in microbial killing. Proteomic studies, during the treatment of *Paracoccidioides* sp. with hydrogen peroxide, depicted the upregulation of ROS detoxification enzymes. These enzymes include catalases, superoxide dismutases, cytochrome c peroxidase and thioredoxin.^8^ Analysis of detoxification mechanisms of *P. brasiliensis* at 6 h post-infection of mouse lung, strongly support the concept that the fungus is exposed to a significant oxidative stress, since cytochrome c peroxidase, superoxide dismutases, thioredoxins and thioredoxin reductase are induced. Histopathology analysis demonstrated that polymorphonuclear cells were in direct contact with *P. brasiliensis* yeast cells, which might account for the fungal response to oxidative stress. In agreement with *in vitro* studies, proteins such as thioredoxin and Cu/Zn superoxide dismutases, accumulated in *P. brasiliensis* yeast cells infecting macrophages.^10^ Enzymes related to detoxification, such as superoxide dismutases, SOD1 (PADG_07418) and SOD2 (PADG_01755) were also accumulated in *P. brasiliensis* yeast at 6 h post-infection. Knockdown mutants for SOD1, although able to infect mouse lung, were unable to disseminate to other organs, suggesting its involvement in intracellular fungus activity.^32^ Cytochrome c peroxidase (PADG_03163) and thioredoxins (PADG_03161, PADG_05504 and PADG_01551) were induced at proteomic levels. Previous studies have described a possible relationship between ROS detoxification and virulence. Cytochrome c peroxidase (CCP) is a potential virulence factor, as demonstrated in studies using the silenced mutant for this gene. CCP silencing reduces *P. brasiliensis* survival during macrophage infection and in yeast cells subjected to *in vitro* nitrosative stress.^9,10^ To maintain cellular redox homeostasis, yeast cells may provide potential reducing for most antioxidant and regulatory enzymes.^33,34^ In this way, activation of the pentose phosphate pathway, which is a source of cellular reducing power in the form of NADPH, reinforces the concept that *P. brasiliensis* yeast cells respond to oxidative stress at 6 h post-infection. Neutralization of free radicals largely depends on NADPH.

Changes in nutrient availability early in the infection process could play a role in the repression of glycolytic genes and upregulation of the glyoxylate cycle. *Paracoccidioides sp* co-cultured with murine macrophages upregulated the transcripts for the glyoxylate cycle-specific enzymes isocitrate lyase and malate synthase, suggested by RT-PCR analyses.^35^ In contrast, in *P. brasiliensis* yeast cells co-cultivated with macrophages,^10^ gluconeogenesis did not appear to play a major role during lung infection, since genes encoding key enzymes as well as cognate proteins in this pathway were not upregulated as determined by proteomics analysis. This finding is in agreement with our results. The stage and site of infection can influence metabolism. Studies on *C. albicans* mutants blocked in various core carbon metabolic pathways demonstrated that the glyoxylate cycle and gluconeogenesis were essential for fungal survival during initial interactions with host immune cells. Later, as the yeast cells colonize, they altered carbon metabolism from non-fermentative to fermentative (i.e., glycolysis).^36^ Similarly, research in *C. neoformans* demonstrated that deletion of pyruvate kinase (*PYK1*) and hexokinases (*HXK1, HXK2*) genes, involved with glycolysis, resulted in impaired fungal survival in the cerebral spinal fluid of rabbits, while displaying persistence in the lungs of mice. *C. neoformans* required gluconeogenesis when it resides in the lung, since the deletion of phosphoenolpyruvate carboxykinase (the *pck1*Δ mutant), resulted in attenuated virulence in a murine inhalation model.^37^ *P. brasiliensis* yeast cells exposed to the lung environment seem to face a carbohydrate-poor environment, which is suggested by increased accumulation of isocytrate lyase. Activation of the glyoxylate cycle may compensate for the lack of carbohydrates in the milieu, as demonstrated for *C. albicans* after phagocytosis by macrophages.^38^

*P. brasiliensis* probably uses multiple carbon sources including lipids, amino acids, and ethanol during infection. At 6 h post-infection, yeast cells upregulated genes and proteins involved with lipid catabolism (e.g., β-oxidation). Similarly, proteomic analysis of *P. brasiliensis* during macrophage infection suggested that yeast cells use fatty acids to survive inside phagocytes.^10^ In the context of metabolic adaptation to the host, we also observed an increase in alcohol dehydrogenase and pyruvate decarboxylase. Pyruvate is most likely supplied by degradation of amino acids. Transcriptional studies from *Paracoccidioides sp* yeast cells recovered from mouse liver suggested that ethanol is produced during infection.^39^ Similarly, we were able to confirm ethanol production by *P. brasiliensis* yeast cells during pulmonary infection. Transcriptome and enzymatic analyses of *Aspergillus fumigatus* demonstrated that this fungus produces ethanol under hypoxic conditions.^40,41^ Ethanol deficient *A. fumigatus* strains promoted an increase in the inflammatory response in infected mice that corresponded to a reduction in fungal burden.^41^

During lung infection, the production and secretion of a serine proteinase was induced in *P. brasiliensis* yeast cells. Immunostaining experiments, demonstrated that, this protein is located intracellularly and in the extracellular milieu. Serine proteinase was not detected in the murine host, after induction of inflammatory response by zymozan, carrageenan and heat-killed fungal cells. The results clearly demonstrated that the polyclonal antibodies do not react with hostt cell proteases. Additionally polyclonal antibodies against this protein reduced the fungal burden of infected animals. There is potential that this serine protease could function as a virulence factor. In *Mycobacterium tuberculosis*, a serine proteinase homolog, mycosin-1, is secreted and contributes to virulence during acute infection.^42^ Additional potential virulence factors can be inferred from our data. Transcriptome data depicted the induction of the Laccase IV gene (PADG_05994), which can be related to increase in fungal virulence. Pathogenic fungi, such as *Paracoccidioides* spp., *C. neoformans, H. capsulatum* and *C. albicans* produce DOPA-melanin via laccase. Melanization appears to contribute to virulence by reducing the susceptibility of melanized fungi to host defense mechanisms and antifungal drugs.^43^

In conclusion, we propose that *P. brasiliensis*, in the initial stages of murine pulmonary infection remodels its carbon metabolism, using preferentially lipids, to survive the harsh host environment. Also, the synthesis of the main components of cell wall can be repressed, putatively enabling the fungus to assemble a cell wall conformation suitable to colonize the host and escape the host immune system. Of special note, an induced serine proteinase is secreted in the murine lung tissue at 6 h post-infection, probably enabling the fungus entering and spreading the lung tissue. To our knowledge, up to know, this is the first report of transcriptional and proteomic data of *P. brasiliensis* upon host infection. We believe that this research provides a successful strategy for recovering *P. brasiliensis* yeast cells from infected lung and opens new perspectives in the study of fungus-host interaction. Additionally this research provides an arsenal of molecules that are expressed *in vivo* that can be relevant to the fungus-host interaction. This study opens new perspectives in the study of fungus-host interaction. Future experiments focusing on kinetics of infection will be performed to establish the metabolic fungus adaptation to the host at different infection times.

## Materials and methods

### Strains and culture conditions

*Paracoccidioides brasiliensis, Pb*18 (ATCC 32069, phylogenetic species S1),^44^ was used in this study. The yeast phase was maintained *in vitro* at 36°C in semisolid BHI medium containing 4% (w/v) glucose. For experiments, the cells were grown during 48 h at 36°C in liquid BHI medium containing 4% (w/v) glucose.

### Ethics statement

Animals were cared according to the Brazilian National Council for Animal Experimentation Control (CONCEA) guidelines. Male BALB/c mice were maintained under controlled environmental conditions, with a temperature of 23–24°C, and a light/dark cycle of 12 h, and provided with food and water *ad libitum*. Male mice aged between 6 to 8 weeks were used for infection with *P. brasiliensis* yeast cells. Animal experimentation was approved by the Ethics Commission on the use of animals at the University Federal of Goiás (CEUA / UFG- protocol numbers 036/2015 and 086/2015). All efforts to minimize animal suffering were made.

### Intranasal infection of mice with *P. brasiliensis* yeast cells

The animals were anesthetized by intraperitoneal injection with a solution containing ketamine hydrochloride (100 mg/kg) (Park, Davis & Company,) and xylazine (10 mg/kg) (Bayer).^45^ Infection was performed by intranasal inoculation of 10^5^ *P. brasiliensis* yeast cells in 100 µL of 0.9% (w/v) NaCl saline solution. For histopathological and immunohistochemical experiments, control group was intranasally^46^ injected with 100ul of saline solution. For transcriptional and proteome experiments, control was obtained with yeast cells growing in liquid medium.

### Histopathological analysis

Histopathological analyses were performed using 3 mice infected via intranasal inoculation and 3 uninfected controls, intranasally inoculate with NaCl saline solution, for 6 and 24 h. Mice were killed in CO_2_ chamber, lungs were harvested, and fixed in 4% (v/v) paraformaldehyde during 24 h. Histological sections of lung were stained with: (a) hematoxylin and eosin (H&E)^12^ and (b) silver-methenamine, in accordance with Gomori-Grocott,^11^ for visualization of yeast cells in the lung tissue. Tissues sections were examined and photographed using Axio Scope A1 microscope. The images were acquired by using software AxioVision (Carl Zeiss AG, Germany).

### Standardization of bronchoalveolar lavage fluid

To confirm that the bronchoalveolar lavage protocol was efficient to remove fungal cells from lung tissue, a histopathological study of the lung was performed after the procedure. This assay was performed with biologic triplicates. Tissues sections were examined and photographed using Axio Scope A1 microscope. The images were acquired by using software AxioVision (Carl Zeiss AG, Germany). The number of yeast cells were counted in 50 different regions of the lung section and compared with lung sections of mouse that were not washed for *P. brasiliensis* removal.

### Obtaining yeast cells from mouse lung

Infected mice were killed in CO_2_ chamber and the procedure of bronchoalveolar lavage was performed by an incision at trachea and insertion of catheter. A total of 3 washes were performed with 1 mL of 0.9% (w/v) NaCl solution. Cells were immediately used for RNA or protein extractions. Control cells were obtained by incubation of *P. brasiliensis* cells in BHI liquid medium at 37°C during 48 h.

For RNA-seq analysis, 9 animals were infected in each experiment, in a total of 3, to obtain each replicate of RNA. An average of 1.5 µg of total RNA was obtained for each triplicate. For proteomic analysis, 12 animals were infected in 3 different assays and equimolar amounts of protein extracts were mixed and used in proteomic analysis. In synthesis, for obtaining the replicates for RNAseq, a total of 27 animals were infected. Considering proteomic analyzes, 36 animals were infected in 3 independent assays.

### RNA-seq analysis

Following recovery of *P. brasiliensis* from lungs, as described above, cell were washed by centrifugation at 4000 g, for 10 min and immediately added of TRIzol reagent (Invitrogen, Carlsbad) to obtain RNA molecules, from 3 independent series of experiments, each one comprising 9 animals. Control cells obtained as described before, were also used to perform RNA extraction. RNA integrity and concentration were assessed by electrophoresis on a 1% agarose gel and by Qubit analyses. The mRNAs from each experimental replicate (9 animals per replicate) and from controls (3 independent culture growth) were purified from total RNA samples, processed and sequenced using Illumina HiSeq 2500 Platform (Tufts Medical School, Boston ).

### Transcriptomic bioinformatics analysis

Approximately 40 million of reads of 100 bp paired-end sequences were obtained for each sample. The resulting FastQ files were analyzed for quality using FastQC (http://www.bioinformatics.babraham.ac.uk./projects/fastqc/). Reads were aligned to the mouse genome reference (GRCm38) to remove any possible host contamination, using Bowtie2 2.2.4 (https://doi.org/10.1038/nmeth.1923). After filtered, the reads were aligned to the reference *Paracoccidioides* genome, *Pb*18, obtained in NCBI under ABKI00000000 identifier. The alignment was done by Bowtie2 program, and each gene was quantified by counting of reads by feature using the function multicov from bedtools package.^47^ Candidates for differentially expressed genes were identified by statistical package DEGSeq, from the Bioconductor repository, using Fisher's test implemented in the package.^13^ Gene features presenting 2.0-fold change cut-off, and p-value < 0.001 were considered regulated and classified.

The biologic processes were obtained using the Pedant on MIPS (http://pedant.helmholtz-muenchen.de) which provides a tool to browse and search the Functional Categories (FunCat) of proteins. The heat maps were generated using Multiexperimental Viewer 4.8 (www.tm4.org/mev).

### Preparation of protein extracts

*P. brasiliensis* yeast cells from infected mouse lung or control cells were collected by centrifugation at 10,000 x *g* during 5 min and washed by addition of 100 μL of RapiGEST (0.2%v/v) (Waters Corp), followed by 3 washes using ultrapure water, and by the addition of extraction buffer (20 mM Tris-HCl pH 8.8; 2 mM CaCl_2_). This suspension was distributed in tubes containing glass beads in equal volume of the cell pellet and the suspension was processed on ice in bead beater equipment (BioSpec) during 5 cycles of 30 sec. The cell lysate was centrifuged at 10,000 x *g* during 15 min at 4°C and the supernatant was quantified for protein content, using the Bradford reagent (Sigma Aldrich, Co.), and bovine serum albumin (BSA) was used as a standard.^48^

### Digestion of protein extracts for nano-ESI-UPLC-MS^E^ acquisition

Enzymatic digestion of proteins was processed, as described previously^10,19,20,24,49^ with some modifications. Briefly, approximately 200 µg of protein was added to 10 µL of 50 mM ammonium bicarbonate pH 8.5, in a microcentrifuge tube. Then 100 µL of RapiGEST™ SF Surfactante (0.2% v/v) (Waters Corporation) was added, and the sample was vortexed and incubated in a dry bath at 80°C for 15 min. Following it was performed the reduction of disulfide bonds with 2.5 µL of 100 mM dithiothreitol (DTT) (GE Healthcare) at 60°C for 30 min, and alkylation of cysteine with 2.5 µL of 300 mM iodocetamide (GE Healthcare) for 30 min at room temperature in the dark. The proteins were subsequently digested with 40 µL of trypsin 0.05 µg/µL (Promega) at 37°C in dry bath for 16h. Trypsin digestion stop and precipitation of RapiGEST were done by acidifying samples whit 40 µL of 5% (v/v) trifluoracetic acid (Sigma-Aldrich). The mixture was incubated for 90 min at 37°C in a dry bath, and centrifuged at 18,000 g at 4°C for 30 min. The supernatants were dried in speed vaccum (Eppendorf). All obtained peptides were suspended in 80 µL of a solution containing 20 mM of ammonium formiate and 150 fmol/µL of PHB (Rabbit Phosphorylase B) (Waters Corporation) (MassPREP™ protein) as internal standard. Samples were transferred to a Waters Total Recovery vial (Waters Corporation).

Nanoscale LC separation of tryptic peptides was performed using a nanoACQUITY™ system (Waters Corporation) equipped with a nanoEase™ 5 µm x Bridge™ BEH130 C18 300 µm x 50 mm precolumn; trap column 5 µm, 180 µm x 20 mm and BEH130 C18 1.7 µm, 100 µm x 100 mm analytical reversed-phase column (Waters Corporation). The peptides were separated in 10 fractions; the gradient elution was performed as follows: 8.7, 11.4, 13.2, 14.7, 16, 17.4, 18.9, 20.7, 23.4 and 65% of acetonitrile/0.1% (v/v) formic acid, with a flow rate of 2000 µL/min. The source was operated in positive ionization mode nano-ESI (+). The lock mass was used for calibration of the apparatus, using a constant flow rate of 0.5 µL/min at concentration of 200 fmol protein GFP [Glu]^1^-Fibrinopeptide B human ([M+2H]^2+^ = 785.8426) (Sigma-Aldrich). Mass spectrometry analysis was performed on a Synapt G1 MS™ (Waters) equipped with a NanoElectronSpray source and 2 mass analyzers: a quadrupole and a time-of-flight (TOF) operating in V-mode. The mass spectrometer was programmed in the data-dependent acquisition mode, in which a full scan in the *m/z* region of 50–2000 was used. Data were obtained using the instrument in the MS^E^ mode, which switches the low energy (6 V) and elevated energy (40 V) acquisition modes every 0.4 s. Samples were analyzed from 3 replicates.

## Data processing and protein identification

The acquired MS raw data were processed using the ProteinLynx Global Server version 2.4 (PLGS) (Waters Corporation). The data were subjected to automatic background subtraction, deisotoping and charge state deconvolution. After processing, each ion comprised an exact mass-retention time (EMRT) that contained the retention time, intensity-weighted average charge, inferred molecular weight based on charge and *m/z*. Then, the processed spectra were searched against *P. brasiliensis* Pb18 protein sequences (Broad Institute; http://www.broadinstitute.org/annotation/genome/paracoccidioides_brasiliensis/Multiome.html) together with reverse sequences. The mass error tolerance for peptide identification was under 50 ppm. The parameters for protein identification included: (i) the detection of at least 2 fragment ions per peptide, (ii) 5 fragments per protein, (iii) the determination of at least 1 peptide per protein, (iv) carbamidomethylation of cysteine as a fixed modification, (v) phosphorylation of serine, threonine and tyrosine, and oxidation of methionine were considered as variable modifications, (vi) maximum protein mass (600 kDa), (vii) one missed cleavage site was allowed for trypsin, (viii) maximum false positive ratio (FDR) of 4% was allowed. For the analysis of the protein quantification level, the observed intensity measurements were normalized with a protein that showed a variance coefficient of 0.025 and that was detected in all replicates (accession number: PADG_04710).

### Proteome bioinformatics analysis

For analysis comparing the ratios between samples recovered from infected lung and control, was used as mathematical model part of the Expression algorithm inside the PLGS software from the Waters Corporation.^50^ The minimum repeat rate for each protein in all replicates was 2. Proteins that presented 50% differences in expression ratios compared with the control were considered to be regulated. Protein tables generated by PLGS were merged, and the dynamic range of the experiment was calculated using the software MassPivot v1.0.1 and FBAT.^51^ The peptide and protein tables were compared using the Spotfire® v8.0 software, and suitable graphics were generated for all data. Microsoft Excel (Microsoft®) was used for table managements. The Uniprot (http://www.uniprot.org) and Pedant on MIPS (http://mips.helmholtz-muenchen.de/funcatDB/) database were used for functional classification. NCBI database was used for annotation of uncharacterized proteins (https://www.ncbi.nlm.nih.gov/). The heat maps were generated using Multiexperimental Viewer 4.8 (www.tm4.org/mev).

### Immunohistochemistry studies

For immunohistochemistry studies, 3 animals were used of test group and 3 animals of control group. Three-µm-sections were obtained from formalin-fixed-paraffin-embedded (FFPE) tissue blocks distended on charged slides (Starfrost White, Sakura Adhesion microscope slide with Cut Edges, ready to use - Dako 9545-1) for hematoxylin-eosin (HE) staining and for immunohistochemistry for microscopic examination. Immunohistochemistry was performed in duplicate, in all slides, which were removed of paraffin by treatment, rehydrated by treatment and washed in distilled water. Endogenous peroxidase was blocked with 8% (v/v) hydrogen peroxide incubation for 20 min. To block non-specific protein, rodent block M (Biocare Medical, #RBM961G) was used, by incubation for 30 min at 37°C.

Antigen retrieval was performed with Rodent Decloaker 1X (Biocare Medical, #RD913L) pH 6.0, for 3 min, at 121°C in pressure cooker (Pascal, Dako Citomation). The mouse polyclonal antibodies anti serine proteinase (anti-PbSAP)^24^ and anti enolase,^25^ were added to the slides at 1:150 dilutions and incubated at 37°C for 2 h, in wet chamber. After, the slides were incubated with Mouse-on Mouse HRP-Polymer (Biocare Medical, #MM620G) for 30 min at 37°C for signal detection, followed by addition of Diaminobenzidine (DAB, Dako, #K3468-1). Sections were counterstained with Mayer's hematoxylin, washed, dehydrated, cleared, coverslipped, and examined by light microscopy. For negative control, the primary antibody was replaced by phosphate buffered saline (PBS 1X), pH 7.4 in slides with *P. brasiliensis.*

### Treatment of animals with immune response inducers

Controls for cross-reactivity of the anti-serine protease antibodies with mouse proteins were performed. Mice were inoculated intranasally, with zymozan, carrageenan or *P. brasiliensis* killed by heating, to induce host immune response. Zymozan (Sigma-Aldrich, Co.), was used to intranasally inoculate mouse at a dose of 0,6mg/g of body weight, as described previously.^26^ Carrageenan (Sigma-Aldrich, Co.) was intranasally inoculated at a dose of 0,05mg/g of mouse body weight, as described previously.^27^

The yeast cells of *P. brasiliensis* were submitted to autoclaving and 10^5^ cells in 100 µL of 0.9% (w/v) NaCl were used to inoculate mouse by intranasal route.

Mice were killed in CO_2_ chamber after 6 hours, lungs were harvested and processed for immunohistochemical analysis, that was performed using polyclonal antibodies to serine proteinase. The assays were performed in biologic triplicates.

### Treatment of mouse with polyclonal antibodies to serine proteinase

Mice were treated with 1mg of polyclonal antibodies to serine proteinase and infected 24 h later with *P. brasiliensis*, with the same protocol described above. After 6 h of intranasal infection, the mice were killed and the lungs were obtained, for further histological and immunohistochemistry analysis.

### Ethanol measurement assay

The concentration of ethanol was quantified by using an enzymatic detection kit according to the manufacturer's instruction (UV-test for ethanol, RBiopharm, Darmstadt, Germany). In the presence of nicotinamide-adenine dinucleotide (NAD), ethanol is oxidized to acetaldehyde by the enzyme alcohol dehydrogenase. This acetaldehyde is quantitatively oxidized to acetic acid in the presence of aldehyde dehydrogenase, releasing NADH, which is determined by means of its absorbance at 340 nm. A total of 10^6^ *P. brasiliensis* yeast cells, obtained from lungs of 15 infected animals, were used to perform the assay. Control condition was also performed by using 10^6^ *P. brasiliensis* yeast cells obtained after incubation in BHI medium, as described previously. Briefly, the cells were lysed using glass beads and bead beater apparatus (BioSpec) in 5 cycles of 30 sec, keeping the samples on ice. The cell lysates were centrifuged at 10,000 x *g* during 15 min at 4°C and the supernatant was used for enzymatic assay according to the manufacturer's instructions. Concentrations of ethanol were obtained in triplicates.

### Evaluation of reduced thiol level

Thioredoxin (Trx) is reduced to dithiol T(SH)_2_ by thioredoxin reductase (TR), in the thioredoxin system. The inhibition of TR decreases total reduced thiol.^52^ Free thiol levels were determined using Ellman's reagent, 5, 5′-dithio-bis-(2-nitrobenzoic acid), DTNB (Sigma Aldrich, Co). A total of 10^6^ *P. brasiliensis* yeast cells, as described above, were obtained from infected mice lung and from control. The cells were centrifuged for 5 min at 8,000 x *g* and lysed by adding 0.5 ml of lysis buffer (50 mM Tris-Cl, 150 mM NaCl, 50 mM ethylenediamine tetraacetic acid [EDTA], pH 7.2), and glass beads in equal volume of the cell pellet. Lysis was performed by vortexing for 3 mixing cycles of 3 min with 1 min intervals, on ice. Cellular debris were removed by centrifugation, and 100 µL of the supernatant was added to 100 µL of 500 mM phosphate buffer, pH 7.5, in each microtiter well, followed by the addition of 20 μl of 1 mM DTNB to each well. Absorbance was measured at 412 nm using a plate reader. Triplicates were obtained for each condition.

## Supplementary Material

KVIR_S_1355660.zip

## References

[1] McEwen JG, Bedoya V, Patiño MM, Salazar ME, Restrepo A Experimental murine paracoccidiodomycosis induced by the inhalation of conidia. J Med Vet Mycol 1987; 25(3):165-75; PMID:3612432; https://doi.org/10.1080/02681218780000231 3612432

[2] Brummer E, Castaneda E, Restrepo A Paracoccidioidomycosis: an update. Clin Microbiol Rev 1993; 6(2):89-117; PMID:8472249; https://doi.org/10.1128/CMR.6.2.89 8472249 PMC358272

[3] Franco M Host-parasite relationships in paracoccidioidomycosis. J Med Vet Mycol 1987; 25(1):5-18; PMID:3553526; https://doi.org/10.1080/02681218780000021 3553526

[4] Bailão AM, Schrank A, Borges CL, Dutra V, Walquíria Inês Molinari-Madlum EE, Soares Felipe MS, Soares Mendes-Giannini MJ, Martins WS, Pereira M, Maria de Almeida Soares C Differential gene expression by *Paracoccidioides brasiliensis* in host interaction conditions: representational difference analysis identifies candidate genes associated with fungal pathogenesis. Microbes Infect 2006; 8(12-13):2686-697; PMID:16962356; https://doi.org/10.1016/j.micinf.2006.07.019 16962356

[5] Tavares AH, Silva SS, Dantas A, Campos EG, Andrade RV, Maranhão AQ, Brígido MM, Passos-Silva DG, Fachin AL, Teixeira SM, Passos GA, Soares CM, Bocca AL, Carvalho MJ, Silva-Pereira I, Felipe MS Early transcriptional response of *Paracoccidioides brasiliensis* upon internalization by murine macrophages. Microbes Infect 2007; 9(5):583-90; https://doi.org/10.1016/j.micinf.2007.01.024 17387029

[6] Parente AF, Bailão AM, Borges CL, Parente JA, Magalhães AD, Ricart CA, Soares CM Proteomic analysis reveals that iron availability alters the metabolic status of the pathogenic fungus *Paracoccidioides brasiliensis*. PLoS One 2011; 6(7):e22810; PMID:21829521; https://doi.org/10.1371/journal.pone.0022810 21829521 PMC3145762

[7] Lima P, de S, Chung D, Bailão AM, Cramer RA, Soares CM Characterization of the *Paracoccidioides* hypoxia response reveals new insights into pathogenesis mechanisms of this important human pathogenic fungus. PLoS Negl Trop Dis 2015; 9(12):e0004282; PMID:26659387; https://doi.org/10.1371/journal.pntd.0004282 26659387 PMC4686304

[8] de Arruda Grossklaus D, Bailão AM, Vieira Rezende TC, Borges CL, de Oliveira MA, Parente JA, de Almeida Soares CM Response to oxidative stress in *Paracoccidioides* yeast cells as determined by proteomic analysis. Microbes Infect 2013; 15(5):347-64; PMID:23421979; https://doi.org/10.1016/j.micinf.2012.12.002 23421979

[9] Parente AF, Naves PE, Pigosso LL, Casaletti L, McEwen JG, Parente-Rocha JA, Soares CM The response of *Paracoccidioides* spp. to nitrosative stress. Microbes Infect 2015; 17(8):575-85; PMID:25841799; https://doi.org/10.1016/j.micinf.2015.03.012 25841799

[10] Parente-Rocha JA, Parente AF, Baeza LC, Bonfim SM, Hernandez O, McEwen JG, Bailão AM, Taborda CP, Borges CL, Soares CM Macrophage interaction with *Paracoccidioides brasiliensis* yeast cells modulates fungal metabolism and generates a response to oxidative stress. PLoS One 2015; 10(9):e0137619; PMID:26360774; https://doi.org/10.1371/journal.pone.0137619 26360774 PMC4567264

[11] Grocott RG A stain for fungi in tissue sections and smears using Gomori's methenamine-silver nitrate technic. Am J Clin Path 1955; 25(8):975-79; PMID:14398663 14398663 10.1093/ajcp/25.8_ts.0975

[12] Huppert M, Oliver DJ, Sun SH Combined methenamine-silver nitrate and hematoxylin & eosin stain for fungi in tissues. J Clin Microbiol 1978; 8(5):598-03; PMID:83328 83328 10.1128/jcm.8.5.598-603.1978 PMC275302

[13] Wang L, Feng Z, Wang X, Wang X, Zhang X DEGseq: an R package for identifying differentially expressed genes from RNA-seq data. Bioinformatics 2009; 26(1):136-38; PMID:19855105; https://doi.org/10.1093/bioinformatics/btp612 19855105

[14] Fernandes L, Araújo MA, Amaral A, Reis VC, Martins NF, Felipe MS Cell signaling pathways in *Paracoccidioides brasiliensis* inferred from comparisons with other fungi. Genet Mol Res 2005; 4(2):216-31; PMID:16110443 16110443

[15] Suresh K, Subramanyam C A putative role for calmodulin in the activation of *Neurospora crassa* chitin synthase. FEMS Microbiol Lett 1997; 150(1):95-100; PMID:9163912; https://doi.org/10.1016/S0378-1097(97)00103-1 9163912

[16] Lenardon MD, Munro CA, Gow NA Chitin synthesis and fungal pathogenesis. Curr Opin Microbiol 2010; 13(4):416-23; PMID:20561815; https://doi.org/10.1016/j.mib.2010.05.002 20561815 PMC2923753

[17] Zhao Y, Su H, Zhou J, Feng H, Zhang KQ, Yang J The APSES family proteins in fungi: Characterizations, evolution and functions. Fungal Genet Biol 2015; 81:271-80; PMID:25534868; https://doi.org/10.1016/j.fgb.2014.12.003 25534868

[18] Murad AM, Souza GH, Garcia JS, Rech EL Detection and expression analysis of recombinant proteins in plant-derived complex mixtures using nanoUPLC-MS(E). J Sep Sci 2011; 34(19):2618-630; PMID:21898799; https://doi.org/10.1002/jssc.201100238 21898799

[19] Murad AM, Rech EL NanoUPLC-MSE proteomic data assessment of soybean seeds using the Uniprot database. BMC Biotechnol 2012; 12:82; PMID:23126227; https://doi.org/10.1186/1472-6750-12-82 23126227 PMC3532185

[20] Li GZ, Vissers JP, Silva JC, Golick D, Gorenstein MV, Geromanos SJ Database searching and accounting of multiplexed precursor and product ion spectra from the data independent analysis of simple and complex peptide mixtures. Proteomics 2009; 9(6):1696-719; PMID:19294629; https://doi.org/10.1002/pmic.200800564 19294629

[21] Pizzatti L, Panis C, Lemos G, Rocha M, Cecchini R, Souza GH, Abdelhay E Label-free MSE proteomic analysis of chronic myeloid leukemia bone marrow plasma:disclosing new insights from therapy resistance. Proteomics 2012; 12(17):2618-631; PMID:22761178; https://doi.org/10.1002/pmic.201200066 22761178

[22] Curty N, Kubitschek-Barreira PH, Neves GW, Gomes D, Pizzatti L, Abdelhay E, Souza GH, Lopes-Bezerra LM Discovering the infectome of human endothelial cells challenged with *Aspergillus fumigatus* applying a mass spectrometry label-free approach. J Proteomics 2014; 97:126-40; PMID:23886778; https://doi.org/10.1016/j.jprot.2013.07.003 23886778

[23] Lima P, de S, Casaletti L, Bailão AM, de Vasconcelos AT, Fernandes G da R, Soares CM Transcriptional and proteomic responses to carbon starvation in *Paracoccidioides*. PLoS Negl Trop Dis 2014; 8(5):e2855; PMID:24811072; https://doi.org/10.1371/journal.pntd.0002855 24811072 PMC4014450

[24] Parente JA, Salem-Izacc SM, Santana JM, Pereira M, Borges CL, Bailão AM, Soares CM A secreted serine protease of *Paracoccidioides brasiliensis* and its interactions with fungal proteins. BMC Microbiol 2010; 10:292; PMID:21080956; https://doi.org/10.1186/1471-2180-10-292 21080956 PMC3000847

[25] Nogueira SV, Fonseca FL, Rodrigues ML, Mundodi V, Abi-Chacra EA, Winters MS, Alderete JF, de Almeida Soares CM *Paracoccidioides brasiliensis* enolase is a surface protein that binds plasminogen and mediates interaction of yeast forms with host cells. Infect Immun 2010; 78(9):4040-50; PMID:20605975; https://doi.org/10.1128/IAI.00221-10 20605975 PMC2937444

[26] Li X, Liu Z, Jin H, Fan X, Yang X, Tang W, Yan J, Liang H Agmatine Protects against Zymosan-Induced Acute Lung Injury in Mice by Inhibiting NF-κB-Mediated Inflammatory Response. BioMed Research Int 2014; 2014:583736; https://doi.org/10.1155/2014/583736 PMC4163488 25243152

[27] - Fröde TS, Medeiros YS Myeloperoxidase and adenosine-deaminase levels in the pleural fluid leakage induced by carrageenan in the mouse model of pleurisy. Mediators Inflamm 2001; 10(4):223-7; PMID:11577999; https://doi.org/10.1080/09629350123332 11577999 PMC1781707

[28] - Bai Y, Wang S, Zhong H, Yang Q, Zhang F, Zhuang Z, Yuan J, Nie X, Wang S Integrative analyses reveal transcriptome-proteome correlation in biological pathways and secondary metabolism clusters in *A. flavus* in response to temperature. Sci Rep 2015; 29(5):14582; https://doi.org/10.1038/srep14582 PMC4586720 26416011

[29] Hall RA Dressed to impress: impact of environmental adaptation on the *Candida albicans* cell wall. Mol Microbiol 2015; 97(1):7-17; PMID:25846717; https://doi.org/10.1111/mmi.13020 25846717 PMC4973840

[30] Böhm L, Muralidhara P, Pérez JC A *Candida albicans* regulator of disseminated infection operates primarily as a repressor and governs cell surface remodeling. Mol Microbiol 2016; 100(2):328-44; PMID:26700268; https://doi.org/10.1111/mmi.13320 26700268

[31] Fang FC Antimicrobial reactive oxygen and nitrogen species: concepts and controversies. Nat Rev Microbiol 2004; 2(10):820-32; PMID:15378046; https://doi.org/10.1038/nrmicro1004 15378046

[32] Tamayo D, Muñoz JF, Lopez Á, Urán M, Herrera J, Borges CL, Restrepo Á, Soares CM, Taborda CP, Almeida AJ, et al. Identification and analysis of the role of superoxide dismutases isoforms in the pathogenesis of *Paracoccidioides* spp. PLoS Negl Trop Dis 2016; 10(3):e0004481; PMID:26963091; https://doi.org/10.1371/journal.pntd.0004481 26963091 PMC4786090

[33] Krüger A, Grüning NM, Wamelink MM, Kerick M, Kirpy A, Parkhomchuk D, Bluemlein K, Schweiger MR, Soldatov A, Lehrach H, et al. The pentose phosphate pathway is a metabolic redox sensor and regulates transcription during the antioxidant response. Antioxid Redox Signal 2011; 15(2):311-24; PMID:21348809; https://doi.org/10.1089/ars.2010.3797 21348809

[34] Ralser M, Wamelink MM, Latkolik S, Jansen EE, Lehrach H, Jakobs C Metabolic reconfiguration precedes transcriptional regulation in the antioxidant response. Nat Biotechnol 2009; 27(7):604-05; PMID:19587661; https://doi.org/10.1038/nbt0709-604 19587661

[35] - Derengowski LS, Tavares AH, Silva S, Procópio LS, Felipe MS, Silva-Pereira I Upregulation of glyoxylate cycle genes upon *Paracoccidioides brasiliensis* internalization by murine macrophages and in vitro nutritional stress condition. Med Mycol 2008; 46(2):125-34; PMID:18324491; https://doi.org/10.1080/13693780701670509 18324491

[36] Barelle CJ, Priest CL, Maccallum DM, Gow NA, Odds FC, Brown AJ Niche-specific regulation of central metabolic pathways in a fungal pathogen. Cell Microbiol 2006; 8(6):961-71; PMID:16681837; https://doi.org/10.1111/j.1462-5822.2005.00676.x 16681837 PMC1472618

[37] Price MS, Betancourt-Quiroz M, Price JL, Toffaletti DL, Vora H, Hu G, Kronstad JW, Perfect JR *Cryptococcus neoformans* requires a functional glycolytic pathway for disease but not persistence in the host. MBio 2011; 2(3):e00103-11; PMID:21652778; https://doi.org/10.1128/mBio.00103-11 21652778 PMC3110414

[38] Lorenz MC, Fink GR The glyoxylate cycle is required for fungal virulence. Nature 2001; 412(6842):83-6; PMID:11452311; https://doi.org/10.1038/35083594 11452311

[39] Costa M, Borges CL, Bailão AM, Meirelles GV, Mendonça YA, Dantas SF, de Faria FP, Felipe MS, Molinari-Madlum EE, Mendes-Giannini MJ, et al. Transcriptome profiling of *Paracoccidioides brasiliensis* yeast-phase cells recovered from infected mice brings new insights into fungal response upon host interaction. Microbiology 2007; 153(Pt 12):4194-207; PMID:18048933; https://doi.org/10.1099/mic.0.2007/009332-0 18048933

[40] Barker BM, Kroll K, Vödisch M, Mazurie A, Kniemeyer O, Cramer RA Transcriptomic and proteomic analyses of the *Aspergillus fumigatus* hypoxia response using an oxygen-controlled fermenter. BMC Genomics 2012; 13:62; PMID:22309491; https://doi.org/10.1186/1471-2164-13-62 22309491 PMC3293747

[41] Grahl N, Puttikamonkul S, Macdonald JM, Gamcsik MP, Ngo LY, Hohl TM, Cramer RA *In vivo* hypoxia and a fungal alcohol dehydrogenase influence the pathogenesis of invasive pulmonary aspergillosis. PLoS Pathog 2011; 7(7):e1002145; PMID:21811407; https://doi.org/10.1371/journal.ppat.1002145 21811407 PMC3141044

[42] Dave JA, Gey Van Pittius NC, Beyers AD, Ehlers MR, Brown GD Mycosin-1, a subtilisin-like serine protease of *Mycobacterium tuberculosis*, is cell wall- associated and expressed during infection of macrophages. BMC Microbiol 2002; 2:30; PMID:12366866; https://doi.org/10.1186/1471-2180-2-30 12366866 PMC131053

[43] Taborda CP, da Silva MB, Nosanchuk JD, Travassos LR Melanin as a virulence factor of *Paracoccidioides brasiliensis* and other dimorphic pathogenic fungi: a minireview. Mycopathologia 2008; 165(4-5):331-39; PMID:18777637; https://doi.org/10.1007/s11046-007-9061-4 18777637 PMC2586806

[44] Matute DR, McEwen JG, Puccia R, Montes BA, San-Blas G, Bagagli E, Rauscher JT, Restrepo A, Morais F, Niño-Vega G, Taylor JW Cryptic speciation and recombination in the fungus *Paracoccidioides brasiliensis* as revealed by genealogies. Mol Biol Evol 2006; 23(1):65-73; PMID:16151188; https://doi.org/10.1093/molbev/msj008 16151188

[45] Green CJ, Knight J, Precious S, Simpkin S Ketamine alone and combined with diazepam or xylazine in laboratory animals: a 10 year experience. Laboratory Animals 1981; 5(2):163-70; https://doi.org/10.1258/002367781780959107 7278122

[46] - Puerta-Arias JD, Pino-Tamayo PA, Arango JC, González Á Depletion of Neutrophils Promotes the Resolution of Pulmonary Inflammation and Fibrosis in Mice Infected with *Paracoccidioides brasiliensis*. PLoS One 2016; 11(9):e0163985; PMID:27690127; https://doi.org/10.1371/journal.pone.0163985 27690127 PMC5045199

[47] Langmead B, Trapnell C, Pop M, Salzberg SL Ultrafast and memory efficient alignment of short DNA sequences to the human genome. Genome Biol 2009; 10(3):R25; PMID:19261174; https://doi.org/10.1186/gb-2009-10-3-r25 19261174 PMC2690996

[48] Bradford MM A rapid and sensitive method for the quantitation of microgram quantities of protein utilizing the principle of protein-dye binding. Anal Biochem 1976; 72:248-54; PMID:942051; https://doi.org/10.1016/0003-2697(76)90527-3 942051

[49] Oliveira LN, Casaletti L, Báo SN, Borges CL, de Sousa Lima P, de Almeida Soares CM Characterizing the nuclear proteome of *Paracoccidioides* spp. Fungal Biol 2016; 120(10):1209-224; PMID:27647238; https://doi.org/10.1016/j.funbio.2016.07.003 27647238

[50] Geromanos SJ, Vissers JP, Silva JC, Dorschel CA, Li GZ, Gorenstein MV, Bateman RH, Langridge JI The detection, correlation, and comparison of peptide precursor and product ions from data independent LC-MS with data dependant LC-MS/MS. Proteomics 2009; 9(6):1683-695; PMID:19294628; https://doi.org/10.1002/pmic.200800562 19294628

[51] Lange C, DeMeo D, Silverman EK, Weiss ST, Laird NM PBAT: tools for family-based association studies. Am J Hum Genet 2004; 74(2):367-69; PMID:14740322; https://doi.org/10.1086/381563 14740322 PMC1181934

[52] Bonfim-Mendonça P, de S, Ratti BA, Godoy J da S, Negri M, Lima NC, Fiorini A, Hatanaka E, Consolaro ME, de Oliveira Silva S, Svidzinski TI β-Glucan induces reactive oxygen species production in human neutrophils to improve the killing of *Candida albicans* and *Candida glabrata* isolates from vulvovaginal candidiasis. PLoS One 2014; 9(9):e107805; PMID:25229476; https://doi.org/10.1371/journal.pone.0107805 25229476 PMC4168232

