# Supplementary material for: Paracoccidioides brasiliensis presents metabolic reprogramming and secretes a serine proteinase during murine infection
Source: Virulence. 2017 Jul 13;8(7):1417–34. doi: 10.1080/21505594.2017.1355660 (PMC5711425; doi:10.1080/21505594.2017.1355660)
Supplement: KVIR_S_1355660.zip [file kvir-08-07-1355660-s001.zip › figure s8.docx]

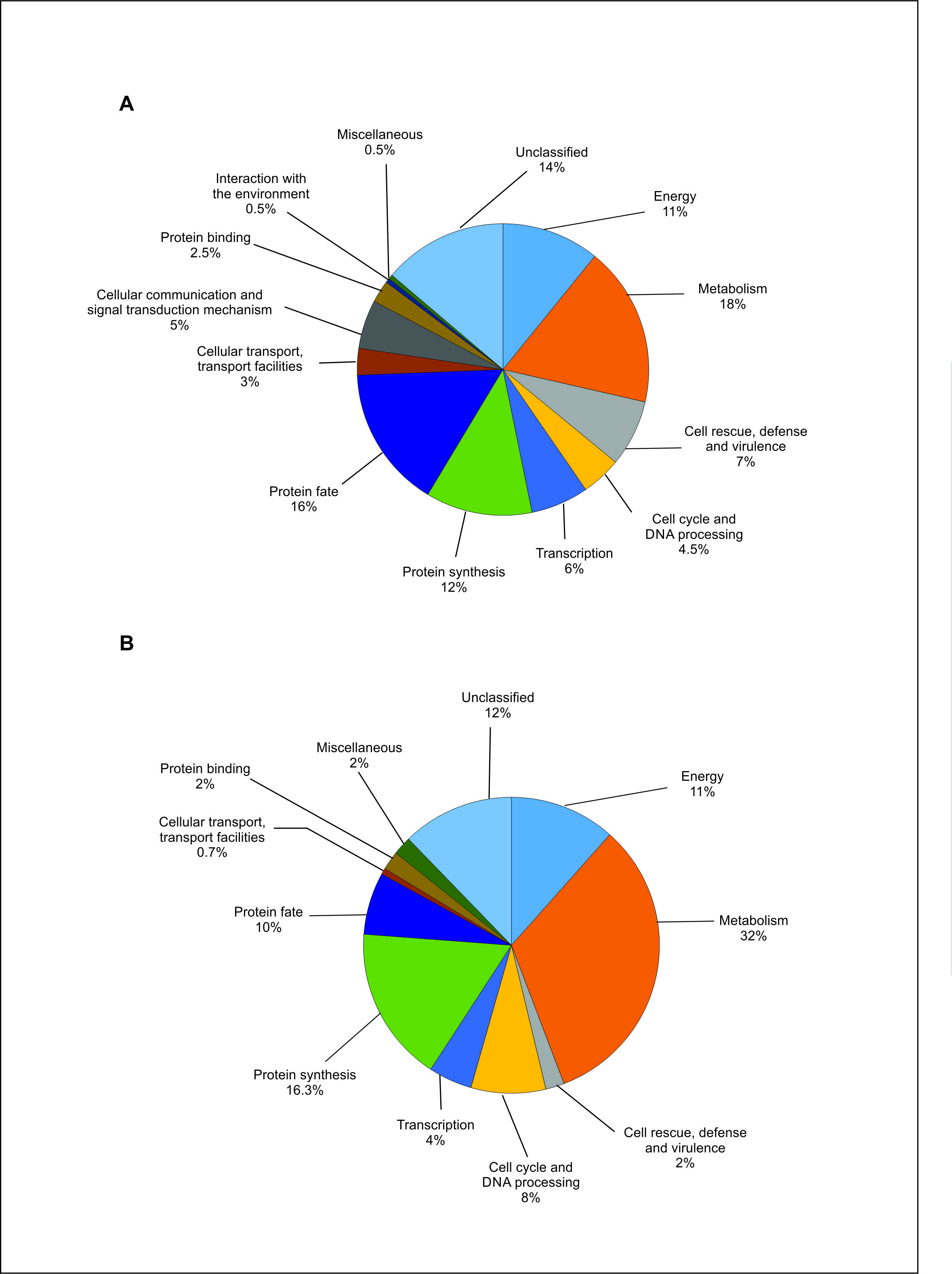


**Supplemental Figure 8: Functional classification and abundance levels of proteins regulated in *Paracoccidioides brasiliensis* during murine lung infection, obtained by NanoUPLC-MSE data.**

**(A)** Biological processes of induced proteins in *Paracoccidioides brasiliensis,,* Pb18, recovered of mouse lung, after 6 hours of infection. The biological processes were obtained using MIPS (http://pedant.helmholtzmuenchen.de/pedant3htmlview/pedant3view?Method=analysis&Db=p3_r48325_Par_brasi_Pb18) and Uniprot databases (http://www.uniprot.org/).

**(B)** Biological processes of repressed proteins in *Paracoccidioides brasiliensis,,* Pb18, recovered of mouse lung after 6 hours of infection. The biological processes were obtained using MIPS (http://pedant.helmholtzmuenchen.de/pedant3htmlview/pedant3view?Method=analysis&Db=p3_r48325_Par_brasi_Pb18) and Uniprot databases (http://www.uniprot.org/).
