# Supplementary material for: Paracoccidioides brasiliensis presents metabolic reprogramming and secretes a serine proteinase during murine infection
Source: Virulence. 2017 Jul 13;8(7):1417–34. doi: 10.1080/21505594.2017.1355660 (PMC5711425; doi:10.1080/21505594.2017.1355660)
Supplement: KVIR_S_1355660.zip [file kvir-08-07-1355660-s001.zip › figure s1.docx]

**
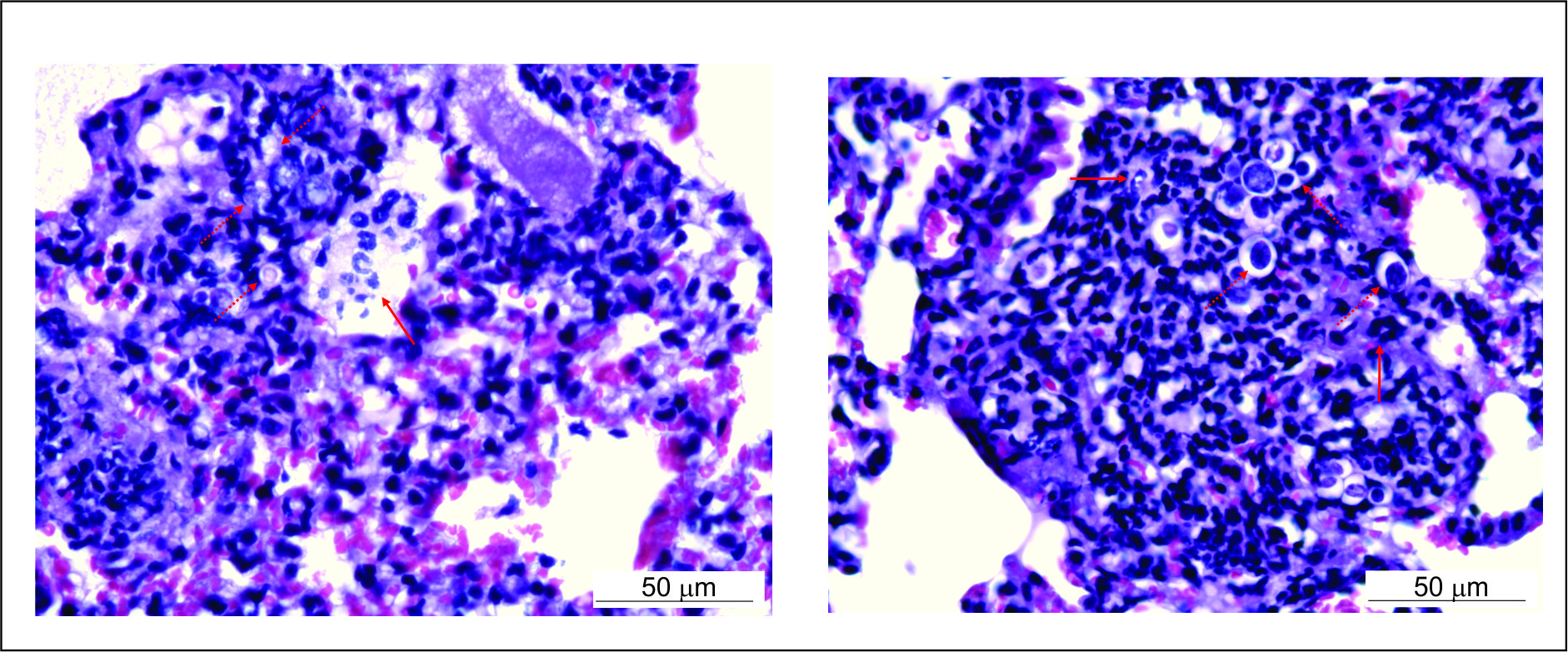
**

**Supplemental Figure 1: Histopathology images of lung sections.** HE staining of the mouse lungs 6 h post-infection with *P. brasiliensis* yeast cells (dotted arrow red). Histopathologic analysis demonstrated exudate, increased cellularity, and neutrophilic inflammatory infiltrate, which was predominantly polymorphonuclear cells (red arrow), and loss of alveolar space.
