# Supplementary material for: Paracoccidioides brasiliensis presents metabolic reprogramming and secretes a serine proteinase during murine infection
Source: Virulence. 2017 Jul 13;8(7):1417–34. doi: 10.1080/21505594.2017.1355660 (PMC5711425; doi:10.1080/21505594.2017.1355660)
Supplement: KVIR_S_1355660.zip [file kvir-08-07-1355660-s001.zip › figure s2.docx]

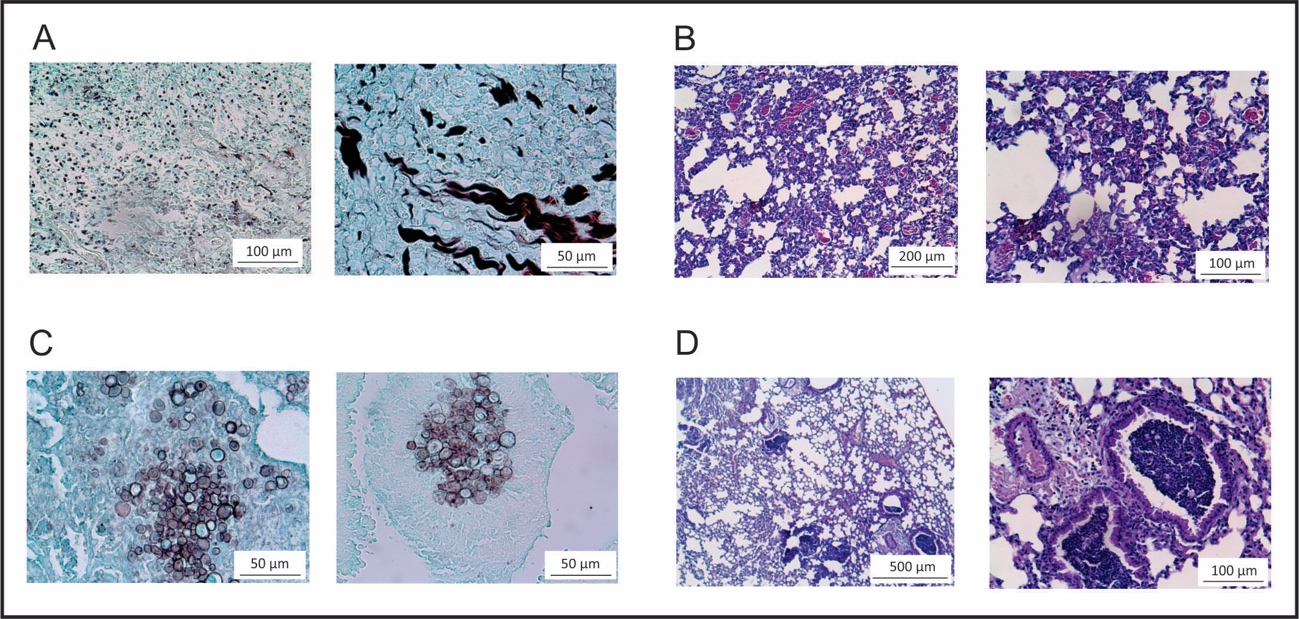


**Supplemental Figure 2: Histopathology images of lung sections.** The animals were euthanized after 24 h post intranasal infection and the lungs were harvested and underwent histopathologic analysis. **A:** Grocott staining in the lung of control animals, which were treated with a saline solution (NaCl 0.9%) **B:** Hematoxylin-Eosin (HE) staining in the lung of control animals **C:** Groccot staining in the lung of animals infected after 24 h. **D:** HE staining in the lung of animals infected after 24 h.
