# Supplementary material for: Paracoccidioides brasiliensis presents metabolic reprogramming and secretes a serine proteinase during murine infection
Source: Virulence. 2017 Jul 13;8(7):1417–34. doi: 10.1080/21505594.2017.1355660 (PMC5711425; doi:10.1080/21505594.2017.1355660)
Supplement: KVIR_S_1355660.zip [file kvir-08-07-1355660-s001.zip › figure s3.docx]

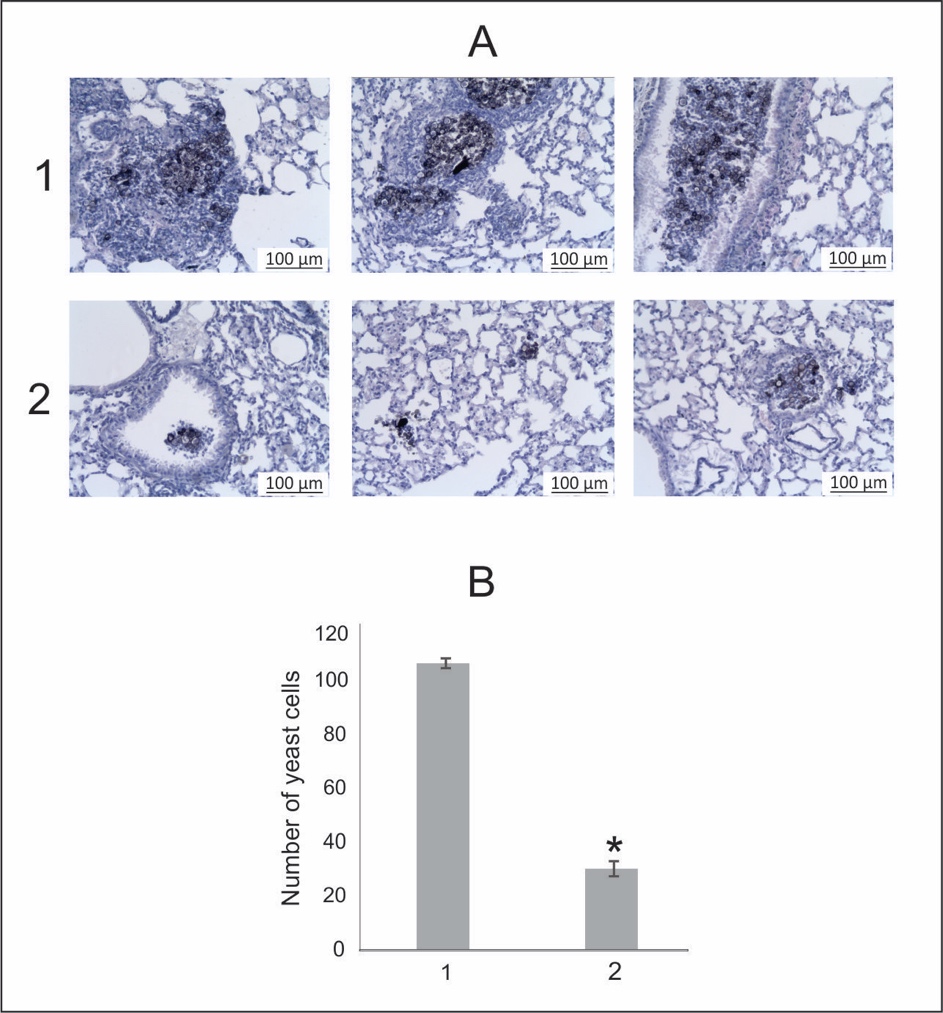


**Supplemental figure 3: Validation of the bronchoalveolar lavage method. (A)** Lung was submitted to histopathological examination. Lung infected with yeast cells of *P. brasiliensis* 6 hours post-infection (1). Infected lung after bronchoalveolar lavage (2). **(B)** Average counts of *P. brasiliensis* yeast cells in lung tissue in 50 fields at 6-hrs post-infection (1) and after brochoalveolar lavage (2).
