# Supplementary material for: Paracoccidioides brasiliensis presents metabolic reprogramming and secretes a serine proteinase during murine infection
Source: Virulence. 2017 Jul 13;8(7):1417–34. doi: 10.1080/21505594.2017.1355660 (PMC5711425; doi:10.1080/21505594.2017.1355660)
Supplement: KVIR_S_1355660.zip [file kvir-08-07-1355660-s001.zip › figure s4.docx]

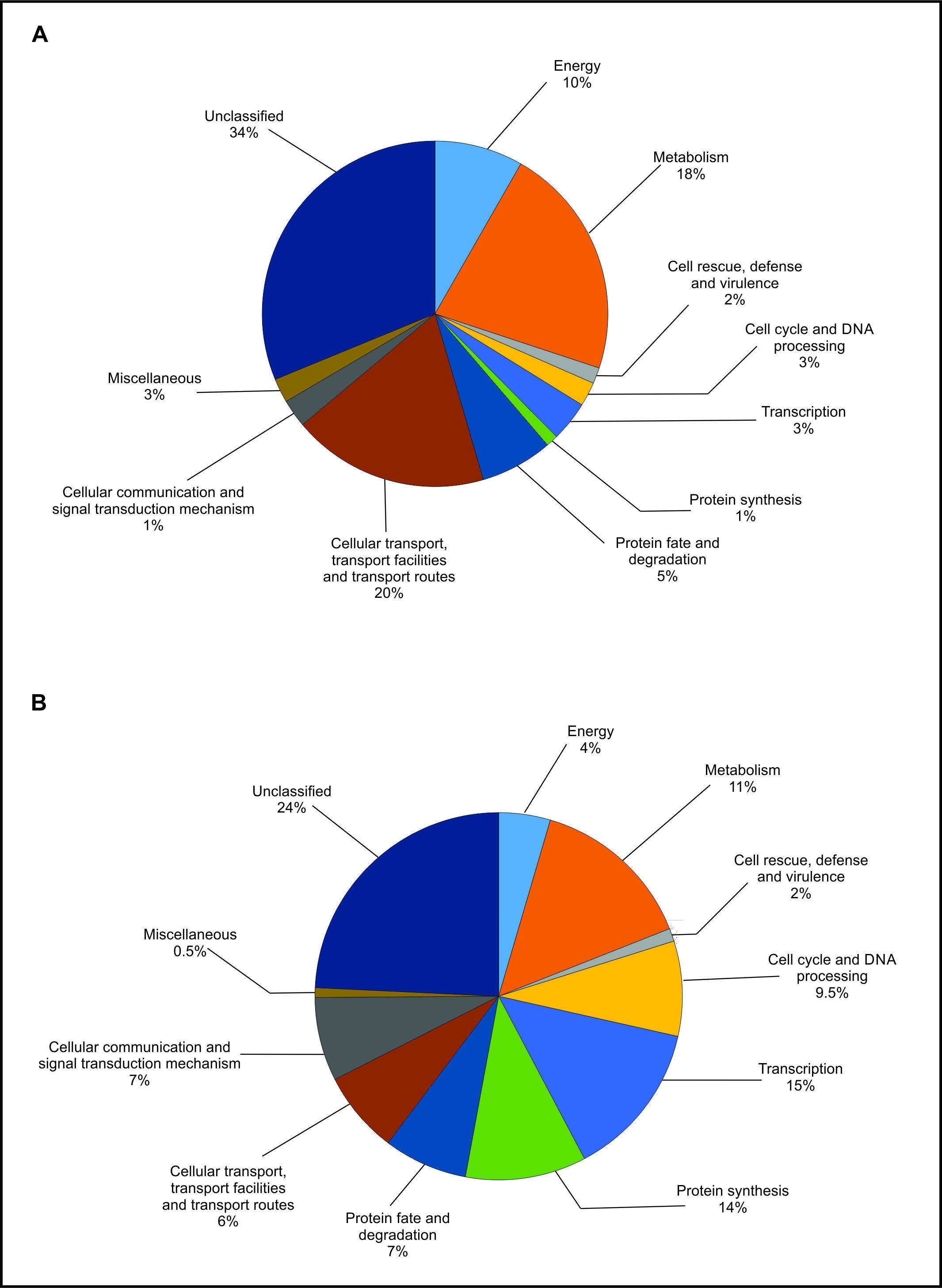


**Supplemental Figure 4- Functional classification of transcripts regulated in *Paracoccidioides brasiliensis* upon 6 h of interaction with the host.**

(A) Biological processes of up-regulated transcripts in *P. brasiliensis*, Pb18, recovered from lung mouse after 6 h of infection. Biological processes were obtained using MIPS (http://pedant.helmholtzmuenchen.de/pedant3htmlview) and Uniprot database (http://www.uniprot.org/).

(B) Biological processes of down-regulated transcripts in *P. brasiliensis*, Pb18, recovered from lung mouse after 6 h of infection. Biological processes were obtained using MIPS (http://pedant.helmholtzmuenchen.de/pedant3htmlview) and Uniprot database (http://www.uniprot.org/).
