# Supplementary material for: Paracoccidioides brasiliensis presents metabolic reprogramming and secretes a serine proteinase during murine infection
Source: Virulence. 2017 Jul 13;8(7):1417–34. doi: 10.1080/21505594.2017.1355660 (PMC5711425; doi:10.1080/21505594.2017.1355660)
Supplement: KVIR_S_1355660.zip [file kvir-08-07-1355660-s001.zip › figure s5.docx]

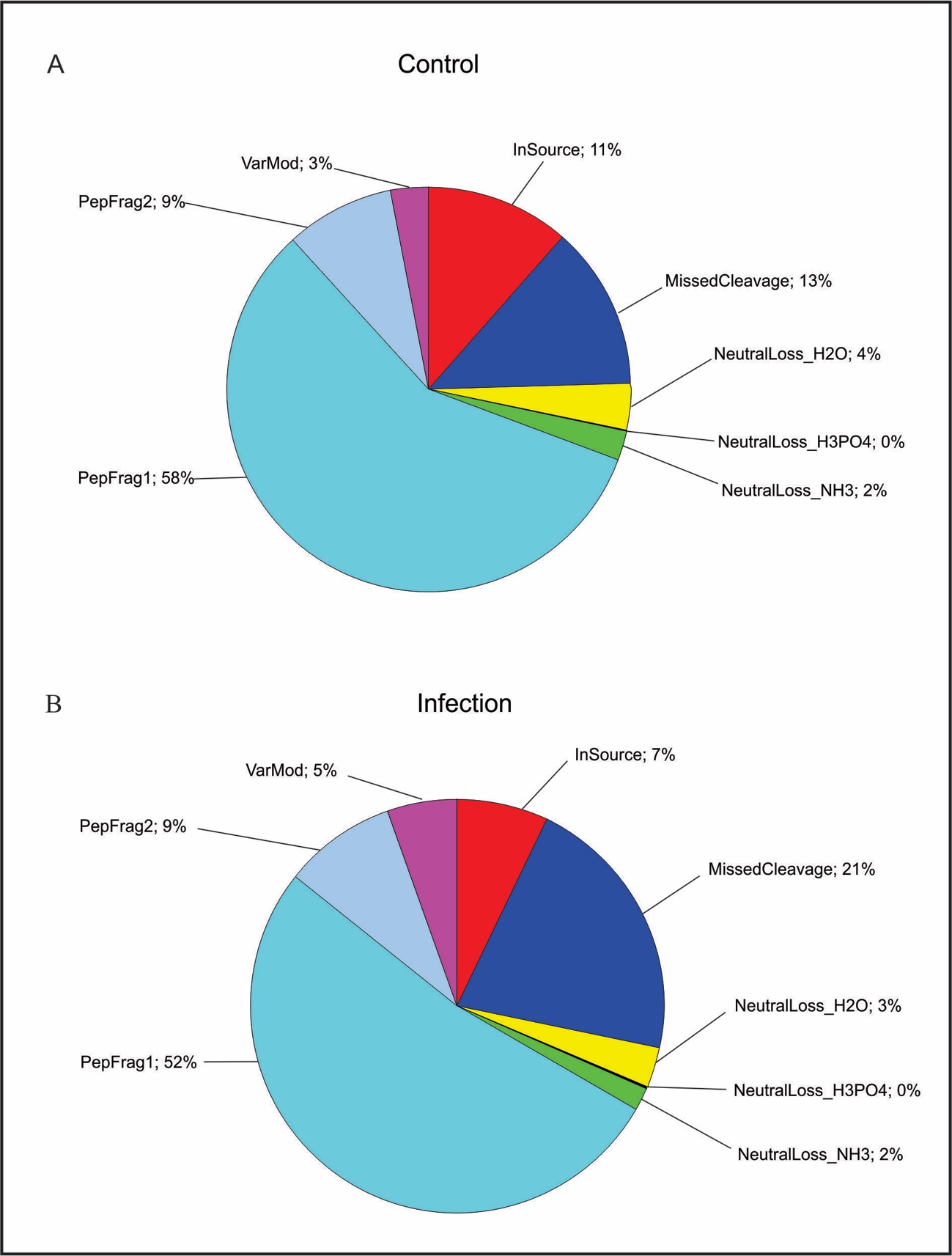


**Supplemental Figure 5: Peptide detection type to control and samples of yeast cells infecting mouse lung.** The pie graph show the percentage of peptides matched against the *P. brasiliensis*  (Pb18) database by PLGS (PepFrag 1 and PepFrag 2), variables modifications (VarMod), fragmentation that occurred on ionization source (InSource), missed cleavage performed by trypsin (Missed Cleavage) and Neutral loss H2O and NH3 corresponding to water and ammonia precursor losses to control (**A**) and infection **(B)** conditions. The SpotFire Decision Site 8.0 v program was used.
