# Supplementary material for: Paracoccidioides brasiliensis presents metabolic reprogramming and secretes a serine proteinase during murine infection
Source: Virulence. 2017 Jul 13;8(7):1417–34. doi: 10.1080/21505594.2017.1355660 (PMC5711425; doi:10.1080/21505594.2017.1355660)
Supplement: KVIR_S_1355660.zip [file kvir-08-07-1355660-s001.zip › figure s6.docx]

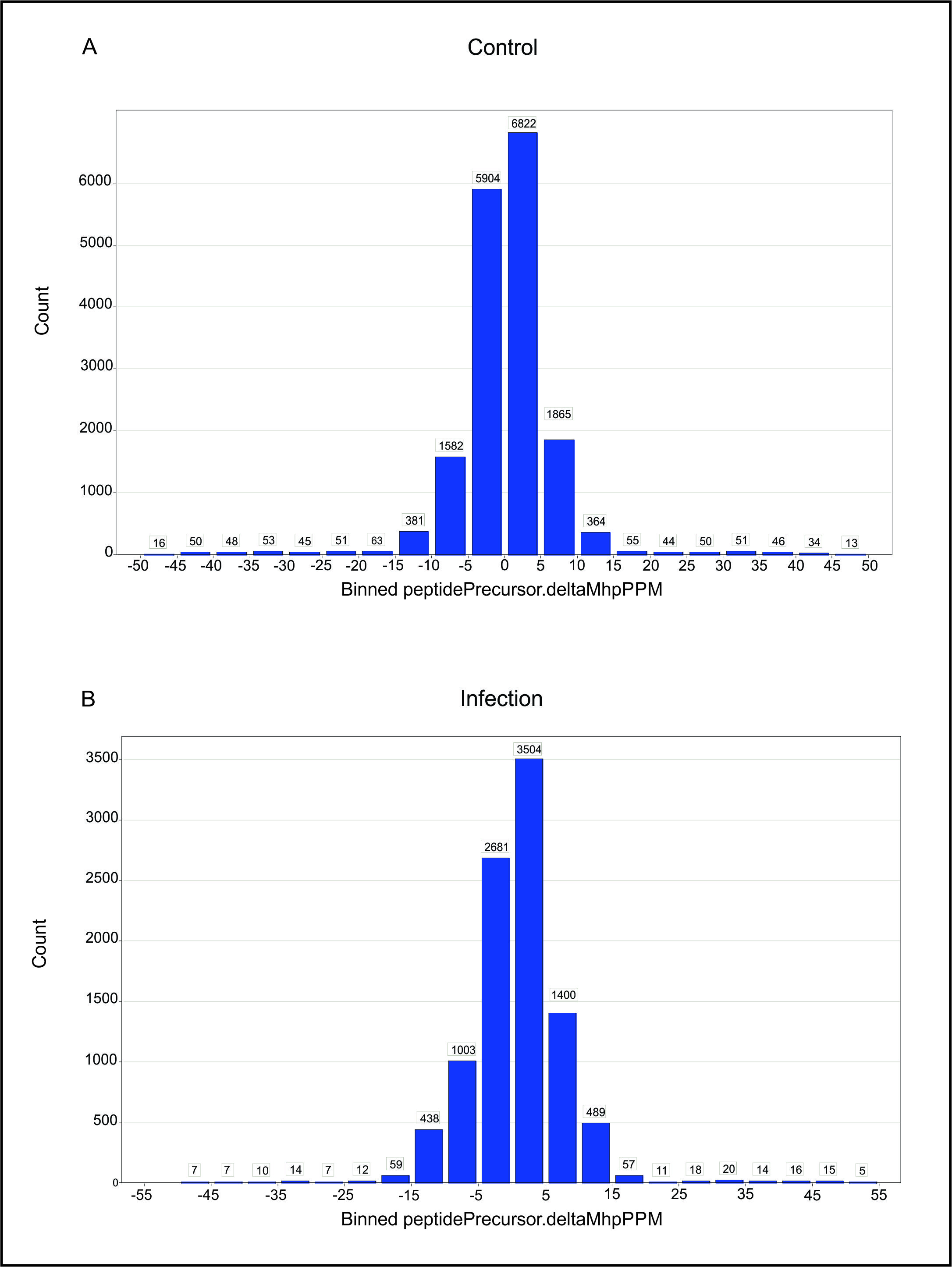


**Supplemental Figure 6: Peptide mass accuracy analyzes**. Peptides data were used to make the bar graph showing the accuracy of mass for peptides in control (A) and infection (B) samples. A total 63.19% and 72.56%, from identified peptides were detected with an error of less than 5 ppm for infection and control conditions, respectively. For error of less than 10 ppm, 87.75% and 92.22% peptides were identified in infection and control, respectively.
