# Supplementary material for: Paracoccidioides brasiliensis presents metabolic reprogramming and secretes a serine proteinase during murine infection
Source: Virulence. 2017 Jul 13;8(7):1417–34. doi: 10.1080/21505594.2017.1355660 (PMC5711425; doi:10.1080/21505594.2017.1355660)
Supplement: KVIR_S_1355660.zip [file kvir-08-07-1355660-s001.zip › figure s7.docx]

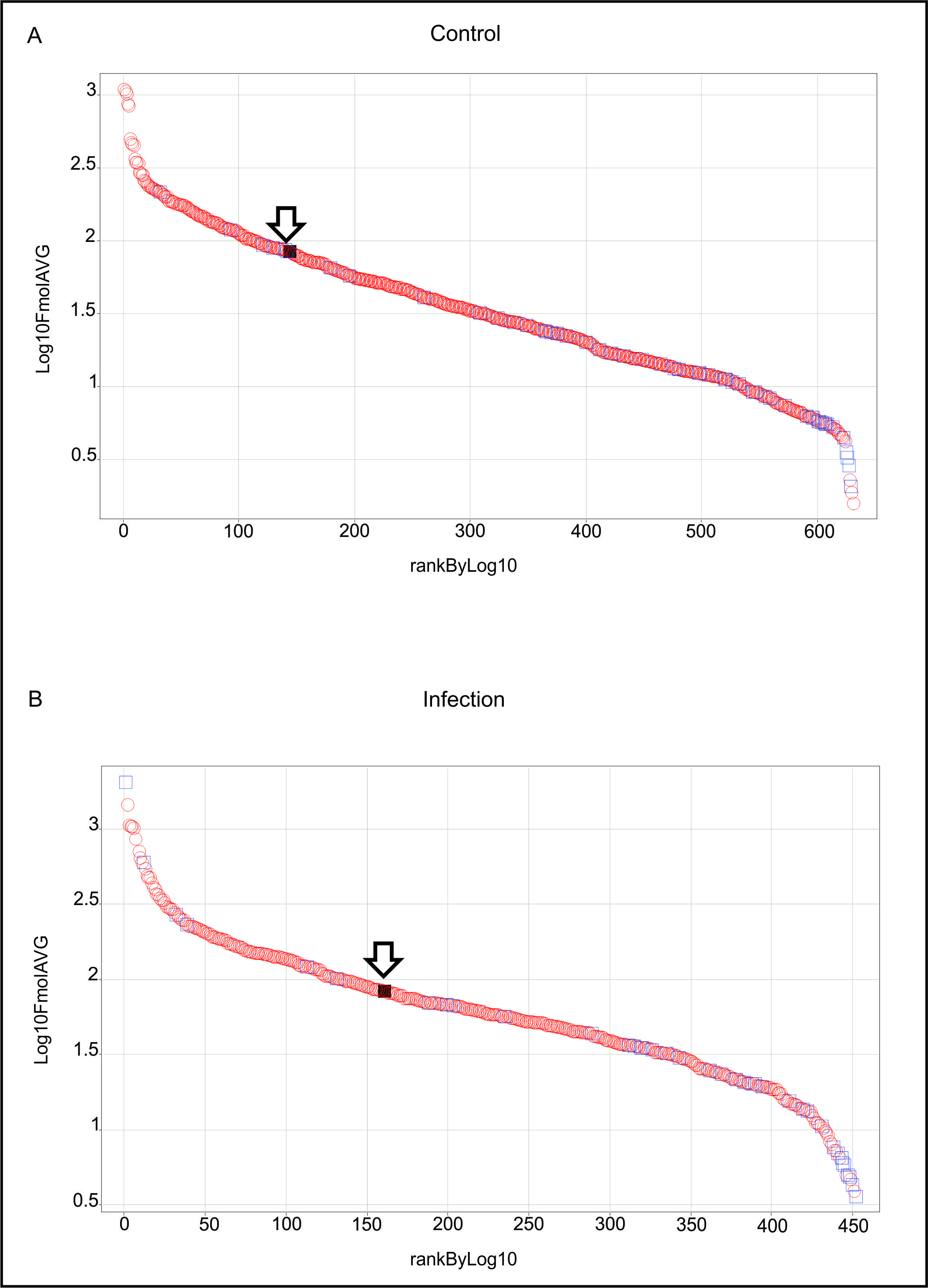


**Supplemental Figure 7: Detection of dynamic range of proteomic analyzes.** The dynamic range the proteomic experiments for each condition was determined. Graphs for control **(A)** and infection **(B)** are shown. Regular and reverse proteins were indicated by red/square, blue/square, colors/shape, respectively. Standard protein is indicated by the arrow. The regular and reverse proteins indicate identified proteins using regular and reverse genomic database from *P. brasiliensis*, respectively. The standard protein was used to normalize the expression data and compare the control and infection related proteins. Our data showed an acceptable quantification to standard protein between the both conditions.
